# From PEGylation to Next-Generation Polymers: Overcoming Biological Barriers—A Review

**DOI:** 10.3390/molecules31040675

**Published:** 2026-02-15

**Authors:** Rizvangul Iminova, Gulzat Berganayeva, Aliya Zhurtbayeva, Lazzat Abdurakhmanova, Almagul Almabekova, Daniil Shepilov, Gulzira Vassilina, Akmaral Nurmahanova, Gulfairuz Kairalapova, Moldyr Dyusebaeva

**Affiliations:** 1 Faculty of Chemistry and Chemical Technology, Al-Farabi Kazakh National University, Almaty 050040/A15E3B4, Kazakhstan; ryzvangul.ymynova@kaznu.edu.kz (R.I.); gulzat-bakyt@mail.ru (G.B.); gufagulfa@gmail.com (G.K.); moldyr.dyusebaeva@kaznu.edu.kz (M.D.); 2 School of Pharmacy, Department of Chemistry, Asfendiyarov Kazakh National Medical University, Almaty 050012/A35B8H9, Kazakhstan; zhurtbaevaaliya@gmail.com (A.Z.); lazzat1984@list.ru (L.A.); 3 Faculty of Biotechnology and Chemical Technologies, Almaty Technological University, Almaty 050012/A05B8Y5, Kazakhstan; vasilina.g@atu.edu.kz; 4 Faculty of Biology and Biotechnology, Al-Farabi Kazakh National University, Almaty 050040/A15E3C7, Kazakhstan; akmaral.nurmahanova@gmail.com

**Keywords:** targeted cancer therapy, nanocarriers, PEG alternatives, low-immunogenic polymers, biodegradable polymers

## Abstract

Poly(ethylene glycol) (PEG) has long stood as the prevailing standard in drug delivery, celebrated for its capacity to enhance solubility, extend circulation, and improve pharmacological performance. Nevertheless, the emergence of anti-PEG antibodies, accelerated clearance, and limited biodegradability increasingly undermine its role as a universal solution. In response, a new generation of polymers has been developed to address these shortcomings, offering the potential to sustain or surpass PEG’s benefits while mitigating immunogenicity, improving biocompatibility, and enabling finer control over therapeutic fate. This review examines current research to articulate a coherent perspective on the replacement of PEG, tracing how advances in polymer design are reshaping the foundations of targeted drug delivery. Taken together, these developments signal not only a corrective to the limitations of PEG but also a broader paradigm shift toward safer, more versatile, and clinically translatable systems that define the next frontier in precision therapeutics.

## 1. Introduction

### 1.1. Methodology of Literature Selection

The literature search was focused on identifying recent research articles that explore advanced and promising alternatives to polyethylene glycol (PEG) for targeted drug delivery systems. The literature search was conducted using Google Scholar and ScienceDirect databases. The search strategy employed combinations of keywords: “Polyoxazoline (POx)”, “polyglycerols”, “zwitterionic polymers”, “polypeptide polymers”, “novel polymer synthesis”, “clinical case”, “usage limitations”, “immunogenicity assessment”, “toxicological evaluation”. Preference was given to studies reporting on polymers with improved bioavailability and prolonged circulation times, as well as evidence of safety, low immunogenicity, and minimal tissue accumulation during long-term use. Particular attention was paid to investigations demonstrating the successful application of the carriers in the treatment of various diseases through both enteral and parenteral administration routes. To ensure relevance and highlight the latest developments in the field, only studies published within the past five years were included, while selected foundational studies were retained to provide context for polyethylene glycol (PEG) and its long-standing use.

### 1.2. PEG Losing Its “Gold Standard” Role

**Figure 1:**
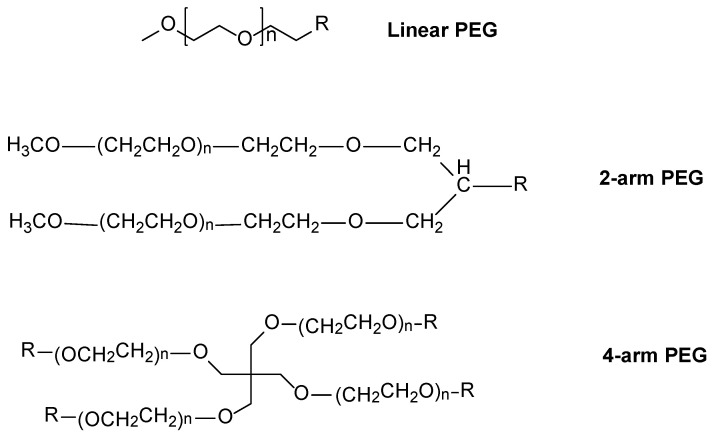
Structural differences in PEG polymers.

Over the past decade, PEG’s privileged status has eroded as reports of anti-PEG antibodies, accelerated blood clearance, and loss of performance across repeated dosing have revealed that PEG (Figure 1) is neither immunologically silent nor universally stable in biomedical environments.

**Figure 2:**
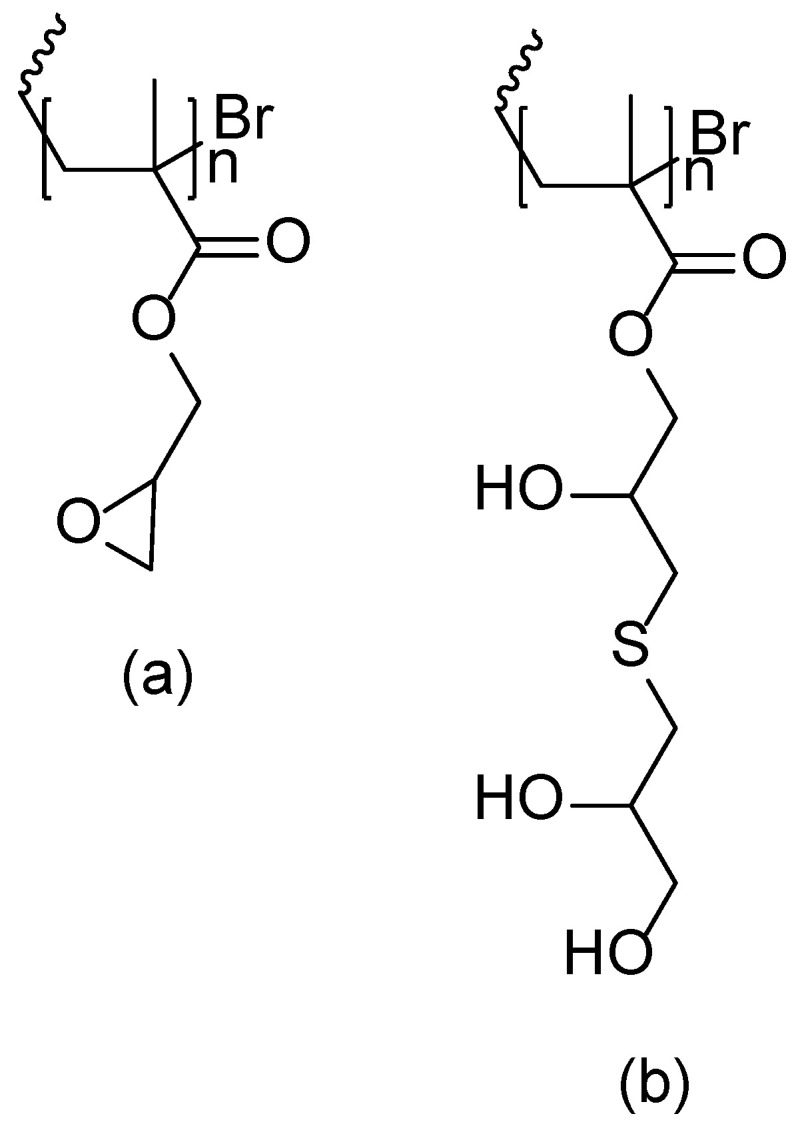
Structures of (**a**) PGMA and (**b**) PEGMA.

Thiol–epoxy click chemistry has been advanced as a PEG-sparing strategy that yields hydroxylated, PEG-like polymers while avoiding classical PEG liabilities. In this approach [1], glycidyl methacrylate (GMA) was polymerized by ATRP to produce PGMA (Figure 2) homopolymers and copolymers with ε-caprolactone (PCL) and PEGMA (Figure 2), followed by high-yield (>80%) post-polymer modification with thiols (thiophenol, thioacetic acid, thioglycerol), with quantitative epoxide conversion confirmed by 1H NMR. Amines reacted poorly, whereas thiols and disulfides were efficiently installed, particularly in the presence of tris(2-carboxyethyl)phosphine (TCEP), which regenerates thiols from disulfides without perturbing epoxide opening. Thioglycerol conjugation afforded poly(2-hydroxy-3-(thioglycerol)propyl methacrylate), a highly hydroxylated analogue exhibiting PEG-like solubility but with higher chemoselectivity and stability; L-cysteine coupling proceeded in >80% yield while preserving free –SH groups as verified by Ellman’s reagent. Cofunctionalization enabled controlled partial substitution (e.g., X% thiophenol, Y% thioglycerol), and sequential installation of thioglycerol and cyclic RGD produced amphiphilic nanoparticles with tunable size distributions suitable for targeting. The work motivates PEG alternatives on the grounds that conventional PEG exhibits oxidative instability, immunogenicity, and low selectivity; here, thioglycerol-derived, highly hydroxylated polymers present a robust substitute that retains bioconjugation potential while mitigating the “PEG dilemma”.

Building on the need for substitutes, a focused appraisal of PEGylation underscores its duality: while PEG reduces renal clearance and immunogenicity and thereby extends circulation half-life, intrinsic features of the synthetic, polydisperse, and non-biodegradable polymer create downstream liabilities. High-molecular-weight conjugates accumulate in the liver (macromolecular syndrome), and although ≈20 kDa PEG is largely renally cleared, larger conjugates display delayed fecal excretion and hepatic retention. The “PEG dilemma” is evident in gene therapy, where the hydrophilic shield stabilizes carriers yet suppresses cellular uptake and endosomal escape, diverting cargo to lysosomal degradation and yielding poor pharmacokinetics for nucleic acids. Clinically, a 40 kDa branched PEG was approved in an aptamer for age-related macular degeneration, but very high mass raises clearance/accumulation concerns; historical products (e.g., ADAGEN, Pegasys, Somavert, PEG-interferons) demonstrate half-life gains alongside immunogenic side effects. Quantitatively, steric masking can be substantial, up to 66% of antibody binding sites shielded in radioconjugates (e.g., DTPA-PEG-C225 with Indium-111 retained only two-thirds of the affinity of the native antibody). PEGylation also enlarges particle size, disturbing biodistribution, while enzymatic cleavage (cytochrome P450, alcohol dehydrogenase) reduces stability. In sum, polydispersity, delayed clearance with hepatic accumulation, steric hindrance, reduced intracellular delivery, and immunogenicity limit PEG’s long-term suitability despite its pharmacokinetic benefits [2].

**Figure 3:**
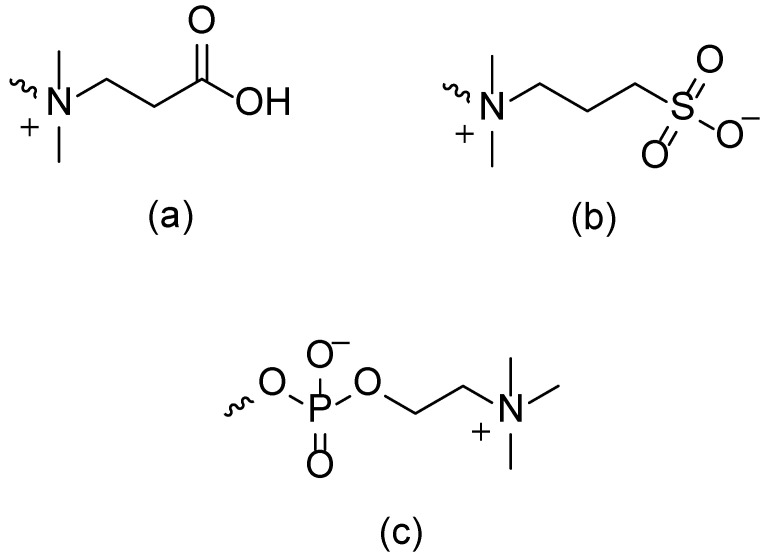
Structures of betaine derivatives: (**a**) carboxybetaine; (**b**) sulfobetaine; (**c**) phosphorylcholine.

A contemporary review consolidates the immunological and structural constraints: up to 72% of individuals harbor detectable anti-PEG antibodies, driving accelerated blood clearance and, in severe instances, hypersensitivity or fatal reactions. PEG conjugation commonly diminishes protein bioactivity via amphiphile-induced shielding; furthermore, linear PEG is limited to two functional termini, constraining drug loading. PEG > 30 kDa accumulates in vivo due to limited degradation, posing toxicological risks, especially in pediatrics; oxidative degradation of linear PEG is too slow to prevent long-term buildup. In contrast, biodegradable zwitterionic polymers based on carboxybetaine, sulfobetaine, and phosphorylcholine (Figure 3) exhibit superior hydration/antifouling and appear immunologically inert (no anti-ZP antibodies). As an example, PLA-SB/PTX (41 mol% sulfobetaine; 6 mol% paclitaxel) forms 19.3 ± 0.2 nm nanoparticles that fully degrade in proteinase K, resist nonspecific fibrinogen adsorption, and enable sustained release; zwitterionic conjugates also enable controlled release of doxorubicin and gemcitabine via acid- or redox-sensitive linkages, directly addressing PEG’s non-degradability and endosomal escape limitations. Thus, degradable zwitterionic systems provide chemically precise, biodegradable alternatives to PEG with enhanced antifouling and loading properties [3].

**Figure 4:**
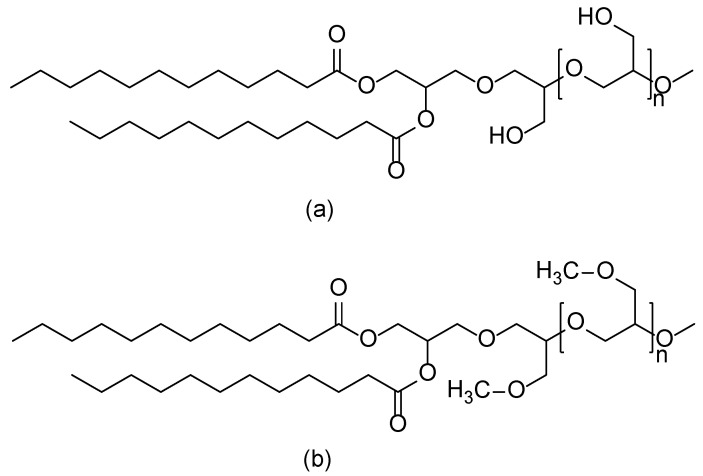
Structures of (**a**) lPG and (**b**) MeOlPG.

Converging evidence in lipid nanotechnology reveals that PEG-lipids (e.g., DMG-PEG2000 in SpikeVax) are immunogenic, eliciting anti-PEG IgM/IgG, accelerated clearance, and anaphylaxis. In antibody-binding assays, PEG-LNPs bound anti-PEG IgG at 1310.55 ng/mL^−1^ (2 mol% PEG) and 816.81 ng/mL^−1^ (0.2 mol%), necessitating a 100-fold dilution to reach background; by contrast, polyglycerol-lipid nanoparticles (lPG-LNPs) exhibited negligible binding (0.50–0.83 ng/mL^−1^). Physicochemically, PEG-LNPs (110.0 ± 6.3 nm, PDI 0.045, encapsulation 98.3 ± 7.5%) contrasted with larger lPG systems (223.1 ± 24.4 nm, PDI 0.105, encapsulation 93.4 ± 8.5%) and MeO-lPG (252.3 ± 14.0 nm, encapsulation 61.9 ± 33.2%); nonetheless, lPG-LNPs maintained comparable colloidal stability over 3 weeks at 4 °C, with ζ-potentials near those of PEG-LNPs (−4.10 to −6.78 mV vs. −3.30 mV). HepG2 assays confirmed >80% viability at 1 µg/mL^−1^ mRNA for all formulations; eGFP mRNA transfection reached ~43% of cells for lPG/MeO-lPG (Figure 4) with median fluorescence 3068.0–3890.0, essentially matching PEG controls. Overall, polyglycerol-lipids preserve stealth, high encapsulation, stable morphology, and negligible antibody cross-reactivity relative to PEG-lipids [4].

Aligned with these observations, substitution of PEG with poly(2-methyl-2-oxazoline) (PMOZ) in LNPs retained high mRNA encapsulation (>95%) while modulating particle size and ζ-potential as PMOZ content rose from 1.0% to 2.5%. In HeLa, 293T, and HepG2 cells, 1.0% PMOZ-LNPs achieved transfection efficiencies comparable to 1.5% PEG-LNPs, whereas THP-1 responses were modest. In PBMCs ex vivo, 1.0% PMOZ and 1.5% PEG induced strong TNF-α, IFN-α, IFN-β, IFN-γ, and IP-10, but higher PMOZ levels attenuated cytokine induction, indicating a negative correlation between PMOZ density and immunostimulation. In vivo immunization (BALB/c; 1 µg rabies glycoprotein mRNA) showed PEG stronger for CD8+ T-cell responses, while 1.0–1.5% PMOZ yielded superior virus-neutralizing titers above the WHO 0.5 IU/mL^−1^ threshold, with 1.5% PMOZ being the highest. These data highlight PEG’s tendency to skew immune responses and support PMOZ as a balanced, PEG-free alternative [5].

**Figure 5:**
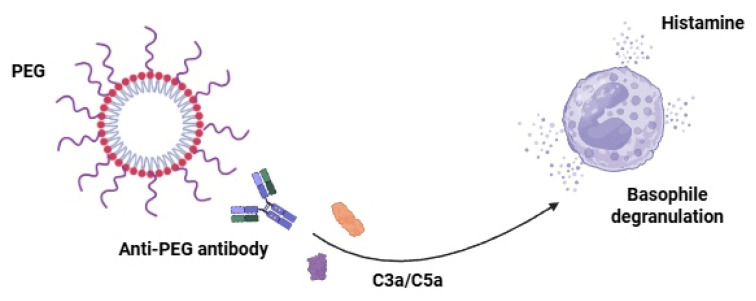
Allergic reaction/hypersensitivity process, caused by PEGylated drugs.

**Figure 6:**
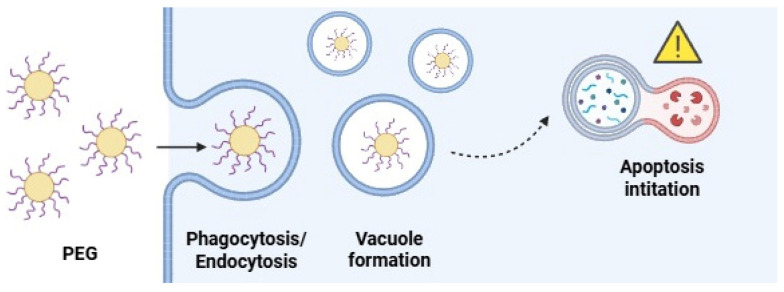
Vacuole formation process induced by PEGylated drugs in phagocytic cells.

**Figure 7:**
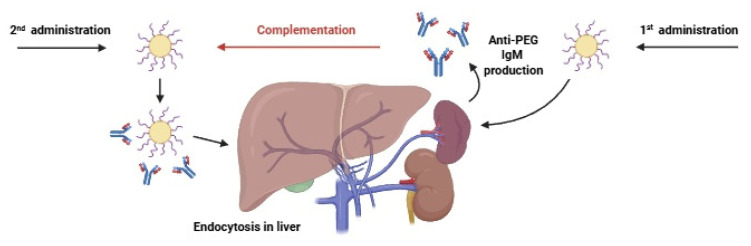
Immunogenicity process of PEGylated drugs.

A comprehensive historical analysis reiterates that, despite transformational impact since the late 1970s (e.g., Doxil, AmBisome, Visudyne), PEGylation entails multiple liabilities: hypersensitivity (Figure 5), cytoplasmic vacuolation (Figure 6), accelerated blood clearance, anti-PEG antibody generation (Figure 7), and organ accumulation for >30 kDa chains (liver/kidney), with oxidative byproducts from lower-MW oligomers posing toxicity. Under heat, radiation, or mechanical stress, PEG degradation becomes unpredictable, complicating drug stability; conjugation can diminish protein affinity and biological activity (waxy solution effects). Commercial activated PEGs are polydisperse, producing ill-defined conjugates and analytical complexity; residual 1,4-dioxane and formaldehyde further raise biomedical concerns. Enumerated alternatives include zwitterionic polymers (poly(sulfobetaine), poly(carboxybetaine), poly(phosphobetaine)), polyglycerols (100–700,000 Da; long circulation yet >100 kDa organ accumulation mitigated by degradable ketal-linked PGs cleared in 1–7 days), poly(2-oxazolines) (PMeOx, PEtOx; PEG-like stealth), poly(amino acids) (poly(L-glutamic acid), PAS (proline/alanine-rich sequence), XTEN; up to 60-fold half-life extension without detectable immunogenicity), PVP (slowly degrading; accumulation risk), and sugar-based systems (HES, dextran, trehalose polymers). Collectively, the case is made for non-PEG stealth materials to resolve immunogenicity, non-degradability, accumulation, and protein activity loss [6].

Head-to-head comparisons of PEG- versus poly(2-ethyl-2-oxazoline) (PEOZ) lipids in LNPs further demonstrate the feasibility of PEG replacement. PEG-LNPs (as in DMG–PEG2000) are immunogenic with preexisting anti-PEG antibodies driving accelerated clearance and rare hypersensitivity. PEOZ-LNPs of similar molecular weight exhibited larger hydrodynamic diameters (145 nm vs. 78 nm) but comparable encapsulation (with efficiency decreasing at higher molar ratios for both). Intravenous mRNA dosing (0.25–1.0 mg/kg^−1^) showed similar liver delivery (endothelial, Kupffer, dendritic cells, hepatocytes) between PEG and PEOZ, while splenic antigen-presenting cells displayed significantly higher uptake with PEOZ (aVHH-positive macrophages and dendritic cells, *p* < 0.01 to *p* < 0.0001). Intramuscular 6 µg mRNA yielded higher expression in splenic dendritic cells and liver endothelial cells for PEOZ. Upon weekly repeat dosing (luciferase mRNA, four injections), PEG-LNPs lost 91% of hepatic expression and 91% of splenic luminescence, whereas PEOZ-LNPs lost only 67% and 71%, respectively; anti-PEG IgM was robust, anti-PEOZ IgM substantially lower, and AST/ALT remained normal. The findings position PEOZ as an effective PEG substitute with superior repeat-dosing performance and lower antibody-mediated clearance [7].

Mechanistic work in SEDDS highlights PEG-specific liabilities at the nano–bio interface: long PEG-chain surfactants (>10 EO units; 30–60% surface fraction; HLB > 13) impart steric and charge shielding that suppress cellular uptake up to 50-fold and traps payloads within the endo-lysosomal pathway (~20-fold higher lysosomal co-localization). Replacing PEG surfactants with polyhydroxy head-group surfactants preserves interfacial behavior while removing intracellular barriers. Polyglycerol (PG)- and alkylpolyglucoside (APG)-based SEDDS produced 35–190 nm nanodroplets with mucus permeation comparable to PEG-SEDDS but markedly enhanced internalization and no lysosomal co-localization; curcumin payloads were maintained with oxidation protection. Polyhydroxy surfaces conferred >3-fold stronger tumor-cell growth inhibition versus PEG-SEDDS by enabling cytosolic delivery. Active surfactant chemotypes included PG4-caprate, PG4-6C, PG4LS, PG6CC, and APG with 1–3 glucoside units; DODAB (cationic) and oleic acid (anionic) served as charge inducers. Formulation tables documented nonionic/anionic/cationic series and showed that PEG removal did not compromise self-emulsification time, dilution robustness, minimal dilution thresholds, or stability in biorelevant media. These quantitative data attribute the PEG dilemma in SEDDS to surface charge shielding and hindered endosomal escape and identify polyhydroxy PG/APG surfactants as active replacements that retain mucus penetration while amplifying cellular uptake and cytosolic drug action [8].

A broader evaluation of PEG surface modification emphasizes oxidative main-chain degradation (toxic byproducts), immunogenicity with anti-PEG IgM and accelerated blood clearance (ABC), and repeat-dose schedule effects. In PEGylated liposomes, cytotoxics such as doxorubicin/mitoxantrone can mask ABC via RES suppression, whereas topotecan does not and elicits strong ABC in animals; clinically, Doxil/Caelyx did not show ABC in patients, but dog data indicate ABC can occur, warranting dose-escalation and anti-PEG IgM monitoring. These constraints frame a stealth-versus-uptake dilemma and motivate alternatives such as poly(2-oxazolines) (PMeOx, PEtOx), which are stealthy, oxidation-resistant, and tunable. Quantitatively, POx triblocks (PMeOx-PBuOx-PMeOx) loaded paclitaxel up to ~45 wt% with potent cytotoxicity against MCF7 ADR and superior in vivo activity versus Cremophor EL PTX; maximum tolerated dose for POx-PTX reached 150 mg/kg^−1^ (vs. 20 mg/kg^−1^ Taxol; 90 mg/kg^−1^ Abraxane). Protein binding was reduced (~38% PTX released to serum proteins), and co-loaded micelles (etoposide/cisplatin) achieved ~52 wt% total loading with wormlike morphology and stronger antitumor effects than single-drug controls; additional actives (SB-T-1214 ~50 wt%, imatinib, panobinostat) demonstrated platform breadth subject to drug–polymer compatibility. Mechanistically, PMeOx/PEtOx coronas form dense antifouling shells akin to PEG but with higher oxidation resistance. PEtOx displayed LCST behavior near ~60 °C, enabling responsiveness; POx minimized complement activation/hemolysis and avoided high-MW organ accumulation typical of PEG. Collectively, PEG’s immunogenicity, oxidative fragility, ABC, and bioactivity shielding contrast with POx attributes of high drug loading, higher tolerated doses, and preserved biocompatibility [9].

The immunochemical basis for clinical failure modes is delineated by quantitative rules for anti-PEG IgM/IgG induction, ABC, and hypersensitivity. Across ≥21 marketed PEGylated drugs (typical PEG 2–40 kDa), ABC exhibits a critical 4–7-day secondary-dose window and has been reproduced in mice, rats, guinea pigs, minipigs, and beagle dogs; clearance depends on lipid dose and schedule. Liposomal PEG density transitions from “mushroom” (less than ~4 mol% PEG2000; ~3–4 nm corona) to “brush” (more than ~9–10 mol%; ~4–10 nm) with higher densities accelerating secondary clearance despite similar IgM induction; chain length modulates immunogenicity (minimum epitope ~750 Da; a 2 kDa chain can bind ~8–15 IgG molecules). Clinical liabilities include HSRs for Doxil/Caelyx, Oncaspar, Neulasta, Macugen, Mircera, Palynziq, Omontys, Krystexxa, and Revolixys, with some products withdrawn. Mechanistic evidence for complement activation-related pseudoallergy (CARPA) includes a pig model where 2K-mPEGylated liposomes induced anti-PEG IgM peaking at days 6–9; re-challenge caused lethal anaphylaxis within minutes with concomitant rises in pulmonary arterial pressure and sC5b-9; in human serum, monoclonal anti-PEG IgG plus complement damaged PEGylated doxorubicin liposomes. These data define PEG-specific risks, including ABC windows, density/length rules, and documented HSR burdens [10].

From a clinical pharmacovigilance perspective, three organ-specific immune-mediated adverse drug reactions implicate PEG and polysorbates as problematic excipients. Benchmarks include drug-related acute pancreatitis at 0.1–2% of all AP cases; diagnostic performance of lymphocyte transformation testing (LTT) shows average sensitivity ~56% and specificity ~94% overall (DILI specificity ~100%, sensitivity ~77%). Case 1 (acute pancreatitis) exhibited positive LTT to lacosamide (SI 4.1) and polysorbate-80 (SI 4.8) with PEG3350 negative; later testing implicated a pantoprazole generic (SI 5.7) consistent with PS80 content. Case 2 (cholestatic DILI) showed negative LTT to amoxicillin–clavulanate but positive T-cell proliferation to PEG3350 and PEG4000 (PEG2000 and PS80 negative), guiding provisional contraindication to PEG ≥ 3000 pending retest. Case 3 (acute interstitial nephritis) demonstrated PS80 positivity by LTT (SI 3.0) and CD69 flow cytometry (SI 5.6). Collectively, the patient-level data reveal size-dependent PEG reactivity and excipient cross-sensitization patterns that complicate safety in PEG-containing targeted formulations and support PEG-free designs [11].

Direct head-to-head bioconjugation of erythropoietin (EPO) further validates the immunological disadvantage of PEG. Antibodies against PEG were detected in 72% of contemporary sera and in 50% of archived sera from the 1970s–1990s, consistent with broad exposure; this pre-existing immunity is associated with hypersensitivity, accelerated clearance, and therapeutic failure. While PEG-EPO (Mircera^®^; FDA-approved 2007) is effective for anemia in kidney disease, infusion reactions and reduced activity arise with antibody formation. Linear polyglycerol (LPG)–EPO, synthesized by site-specific SPAAC, retained full glycosylation, remained stable in human serum beyond 24 h, and matched PEG-EPO bioactivity in TF-1 proliferation assays; unlike PEG, LPG offers hydroxyl functionality for further derivatization and lacks known antibody prevalence, highlighting a practical, less immunogenic alternative [12].

Finally, a targeted literature review of human experience aggregated 29 studies and found that 18 reported adverse safety outcomes with PEGylated proteins (hematologic toxicity; hepatic enzyme elevation; injection-site reactions; arthralgia; nausea; infections; grade 3/4 events; discontinuations), 15 documented immunogenicity (pre-existing and treatment-emergent anti-PEG), and seven reported pharmacologic consequences (increased clearance; reduced activity). Quantitatively, PEG-IFN plus ribavirin doubled neutropenia risk versus IFN (risk ratio 2.15–2.25, *p* < 0.0001), increased thrombocytopenia > 2-fold (RR 2.28–2.63, *p* < 0.0001), and caused leukopenia in up to 56% versus 23.5% with IFN; in melanoma, granulocytopenia and leukocytopenia were three-fold and five-fold more frequent with PEG-IFN (*p* < 0.0001). Hepatic toxicity was higher with PEG-asparaginase versus native enzyme (grade 3–4 liver toxicity 11% vs. 3%, *p* = 0.009). In type 1 diabetes, peglispro produced ALT ≥ 3× ULN in 4.5% versus 0.7% with glargine (*p* = 0.041) and ~30% ALT elevation at 26–78 weeks. PEG-asparaginase revealed pre-existing anti-PEG IgG in 13.9% and IgM in 29.1%, with therapy further boosting IgG/IgM that correlated with reduced activity (OR IgG 2.06, 95% CI 1.44–2.96, *p* < 0.001; OR IgM 1.65, 95% CI 1.27–2.15, *p* < 0.001). Large pediatric ALL cohorts reached anti-PEG positivity in up to 100% during intensification, with accelerated clearance and hypersensitivity despite lower allergic rates than native enzyme. These consolidated human data frame PEG-specific safety and efficacy liabilities highly relevant to drug-targeted delivery [13].

At present, a wide range of pharmaceutical products, approved by regulatory authorities such as the U.S. Food and Drug Administration (FDA), incorporate PEG as part of their delivery systems (Table 1). These PEG-containing formulations span a broad spectrum of indications. It is important to recognize that such adverse effects do not represent the entirety of clinical experience. In fact, PEGylated carriers have repeatedly shown favorable stability and life-saving efficacy in critical clinical settings where rapid and reliable drug performance is essential. Nevertheless, the exploration and development of alternative polymeric carriers with reduced immunogenicity and more predictable regulatory profiles is both timely and necessary for next-generation drug delivery platforms.

### 1.3. Biological Barriers in Drug Delivery

Although synthetic polymers remain foundational in drug delivery and tissue engineering, their intrinsic heterogeneity, manifested as broad dispersity, random sequence distribution, uncontrolled degradation pathways, and poorly regulated microenvironments, creates design blind spots that lead to unpredictable release kinetics, batch-to-batch inconsistency, and biologically irritating degradation profiles.

Sequence control in polyesters has emerged as a key lever to mitigate acidity-driven failure modes in PLGA carriers: local acidity and inflammatory risk are governed by monomer order rather than solely by the lactic:glycolic ratio. Sequenced copolymers (poly LG and poly LracG; Mn 21.4–29.7 kDa; Tg 49–50 °C) formed 50–150 µm microparticles that maintained higher internal pH and delayed percolation of acidic by-products relative to composition-matched random PLGAs (PDLGA-50, PLLGA-50, PDLGA-65; Mn 22.4–28.9 kDa; Tg 47–48 °C). Two-photon pH mapping (LysoSensor Yellow Blue DND-160, calibrated pH 2.83–6.44) showed random 50:50 PLGA developed widespread acidic microclimates (≈pH 4.5 by days 9–11) with acid-filled pores and structural loss by day 14, whereas sequenced poly LG and poly LracG exhibited minimal pH drift and fewer acidic pockets through 21–59 days; literature values cited indicate random PLGA interiors can reach pH 1.5–3.5 by 15–28 days, jeopardizing acid-labile payloads. Subcutaneous implantation corroborated that alternating-sequence poly LG elicited a lower granulomatous foreign-body response than racemic random 50:50 PLGA, establishing a design rule that sequence control stabilizes the internal pH and reduces tissue reactivity without added neutralizers or steroids [14].

**Figure 8:**
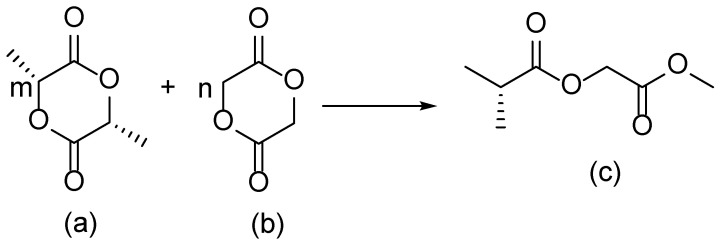
Synthesis of PLGA: (**a**) L-lactide; (**b**) glycolide; (**c**) PLGA.

Drug–polymer microenvironments then determine real-world release behavior in PLGA implants (Figure 8). In Resomer RG 502H (50:50) devices with dihydropyridines, manufacturing reduced PLGA Mw by ≈5–7% and Tg from ≈37 °C to 24.6 °C; drugs acted as plasticizers (5 wt% nifedipine, Tg 16.4 °C; 5 wt% nicardipine, Tg 18.4 °C). Water-uptake factors (WUF) climbed to 11-fold (5% nifedipine) and nine-fold (10% nifedipine) by day 14 with erosion to 52.2 ± 1.8% and 61.6 ± 4.4% polymer loss, leaving ≈29% polymer at day 21; nicardipine implants took up more water early (protonation at acidic pH) reaching WUF ≈ 18 by day 12 and diverging to 32.7 ± 3.7 (5%) versus 5.1 ± 1.2 (10%) by day 21 as the higher-loaded matrix largely depleted. Counterintuitively, lower nifedipine load (5%) delivered both higher relative and higher absolute release (23.3% day 7; 68.5% day 14; 84.5% day 21) compared with 10% (<5% day 7; 25.8 ± 9.4% day 14; 30.2 ± 1.9% day 21). For nicardipine, profiles were initially similar, with 5% surpassing 10% after day 14 and absolute release from 5% matching or exceeding 10% later. Microscopy, PXRD, and content assays indicated amorphous-to-crystalline transitions inside degrading PLGA and payload supersaturation with precipitation (polymer-phase drug rising from nominal 10% to ~20% for nifedipine and >50% for nicardipine at high load), which slows dissolution per Noyes–Whitney and decouples erosion from release, implicating lower initial loading as more effective over ~2–3 weeks for poorly soluble basic drugs by avoiding precipitation-limited plateaus [15].

Mechanistic syntheses of PLGA delivery establish that payloads are released via diffusion through water-filled pores, diffusion through polymer, osmotic pumping, or pure erosion, with rates governed by rapid water uptake, autocatalytic hydrolysis, heterogeneous bulk erosion, pore formation/closure, polymer–drug and drug–drug interactions, and intra-matrix acidification that can depress pH to 1.5–4.7. Transport resistances are modulated by Mw (often <50 kDa), lactide:glycolide ratio (50:50–100:0), end-group capping, semi-crystallinity, and device geometry/size that amplify acid gradients; environment (temperature, stirring, osmolality, buffer capacity) further tunes kinetics. Rate-controlling phenomena include pore closure near Tg shifts as Mw drops and oligomer dissolution around Mw ≈ 1100 Da; dissolution-plus-diffusion, polymer relaxation, crack formation, and osmotic pumping all contribute. Additives (e.g., divalent cations as pore-forming catalysts; basic anions for acid neutralization) can modulate profiles, while frequent medium exchange prevents external acidification; conversely, large constructs and low-sink settings intensify internal acid build-up to pH 1.5–3.5 within weeks. Collectively, PLGA’s core problem for drug targeting is a dynamic, acidity-driven microenvironment that undermines protein stability and predictability over hours to months as a function of Mw, L:G, and device scale [16].

At the protein formulation level, multiple stressors jeopardize native structure during PLGA processing, storage, and release. Water/organic interfaces and shear/cavitation during emulsification induce adsorption, unfolding, and aggregation (EtAc generally milder than MC with protein-specific exceptions); suspension loading alleviates interfacial damage but demands lyoprotectants (trehalose, mannitol) to preserve proteins such as rhGH and enable fine BSA suspensions. Spray-drying imposes brief high-temperature stress, yet, in cryogenic variants, preserved month-long rhGH bioactivity. Storage instabilities include moisture-accelerated hydrolysis and acidification (e.g., at 40 °C/75% RH for one month, insulin deamidation and covalent dimers rose; intra-particle pH declined to ~3.8 within three weeks). Release commonly features a day-1 burst followed by incomplete recovery of native protein owing to acidification and polymer–protein interactions; intra-microparticle pH between 1.5–4.7 has been directly measured and linked to hydrolysis, deamidation, and antigen degradation. Electrostatic vs. hydrophobic binding to PLGA can be deconvoluted (NaCl for electrostatics; GdnHCl for non-covalent aggregates; SDS for hydrophobic adsorption/aggregates). Representative outcomes include early lysozyme retention via electrostatics with progressive non-covalent aggregation; carboxymethylated BSA and BSA non-release dominated by adsorption extractable with SDS; L-asparaginase losing all activity in capped PLGA by two weeks but retaining ~50% in uncapped PLGA at three weeks. Analytical artifacts (e.g., high-g centrifugation “squeezing out” protein; solvent-induced structural changes) can misstate release or integrity. These data map the most problematic stresses and most active stabilizers/process parameters across rhGH, rhEPO, lysozyme, insulin, BSA, toxoids, PVA emulsifier, MC/EtAc solvents, and excipients such as hydroxypropyl-β-cyclodextrin, trehalose, mannitol, and zinc [17].

Beyond degradable polyesters, drug-eluting metallic sutures confront uncontrolled corrosion, burst release, infection risk, and inflammation. A polyelectrolyte multilayer (PEI/PAA) infused with Cocos nucifera extract on AZ31 Mg stitch wires increased tensile strength (~240 to ~267 N) and markedly slowed degradation in simulated GI fluids (uncoated fully degraded by day 14; coated retained 22–31% mass). The coating enabled pharmaceutical-grade loading with 67.54 ± 0.32% encapsulation efficiency and biphasic elution following Gallagher–Corrigan kinetics (R^2^ = 0.99995; RMSE = 0.89%): an 18.22% burst at 2.1 h followed by matrix-erosion-driven release to 120 h. Hemocompatibility (<1% hemolysis), cytocompatibility (>85% HDFa viability), and robust antibacterial activity (zones up to 24 mm against *E. coli*, *S. aureus*, *K. pneumoniae*) were achieved via medium-chain fatty acids (lauric, capric), monolaurin, and phenolic antioxidants (caffeic, ferulic) that disrupt membranes and dampen inflammation; rat wound histology showed accelerated closure, fibroblast proliferation, and organized collagen around coated implants. The PEI-anchored PAA matrix thus addresses canonical polymer-coating problems for drug-target implants, premature corrosion, alkaline microenvironments, hydrogen evolution, infection susceptibility, and dose dumping, by regulating interfacial charge transport and diffusion [18].

**Figure 9:**
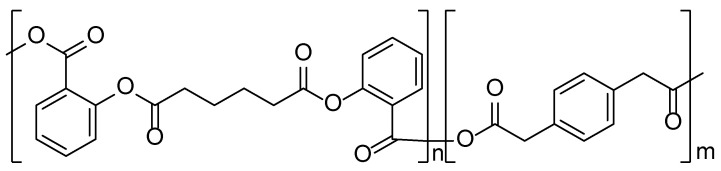
Structure of SAA-co-PA.

For topical retinoids, polymer-controlled precision remains the limiting factor. A salicylic-acid-derived poly(anhydride-ester) (SAPAE) microsphere platform co-delivered retinol (diffusion-controlled; 0.5–1.5 wt% load; particles 8.6 ± 0.8 µm when loaded) and covalently bound salicylate (surface-erosion; 54–77 wt% dictated by composition), with pH-responsive kinetics faster at pH 9.0 than at 7.4 or 5.5. The most active copolymer, SAA-co-PA (with p-phenylenediacetic acid), minimized retinol burst while sustaining salicylate elution and maintained processable thermal properties (Tg 28–48 °C; Td 257–282 °C; comonomer incorporation: PA 7.66%, FA 11.9%, AA 52.5%). Process robustness (60–70% yields; residual solvent below NMR detection) and kinetic modeling (zero-, first-order, Higuchi, Korsmeyer–Peppas, Hixson–Crowell, Baker–Lonsdale, Gallagher–Corrigan) with Flory–Huggins correlations validated mechanism assignments, solving erratic burst and subtherapeutic tail in dual-active topical systems and nominating SAA-co-PA (Figure 9) for synchronized retinoid–salicylate delivery [19].

Quality-by-design control of PLGA attributes is crucial because hydrolytic autocatalysis, heterogeneous bulk erosion, and acidification drive instability and variability. Release kinetics and stability hinge on Mw, intrinsic viscosity, monomer ratio, blockiness, and end-cap chemistry; polystyrene-standard GPC can misestimate PLGA Mw by up to ~70% versus MALS, complicating critical-quality-attribute control. Clinical products exemplify the diversity: Vivitrol^®^ microspheres contain 33.70 wt% naltrexone with 66.30% of a 75:25 PLGA; Lupron Depot^®^ employs 10% leuprolide acetate with 1.7% gelatin in 75:25 PLGA; Eligard^®^ 7.5 mg uses 50:50 acid-capped PLGA (82.5 mg polymer) while other strengths switch to 75:25 hexanediol-capped PLGA, choices that shift degradation and release windows. End-cap effects alone can delay degradation 4–6 weeks (ester- vs. acid-capped), raising risks of lag-phase underexposure or burst-phase toxicity in local targeting. Higher glycolide accelerates hydrolysis and acid microclimates; lower Mw accelerates burst/erosion; greater blockiness amplifies G–G cleavage and broadens Mw distributions, undermining predictable transport. FDA-listed regimens from weekly to 6-month intervals reflect these levers; for example, Eligard^®^ 45 mg achieves six-month exposure via copolymer selection. Even 5 kDa Mw shifts or 5% L:G changes alter risperidone profiles; level-A IVIVC is achievable only with tightly constrained processes and polymer lots. Stimuli-responsive or ligand-modified hybrids may improve spatiotemporal targeting but inherit PLGA’s acidification/heterogeneity risks, motivating sequence control, buffering, or hybrid strategies [20].

Oxidative-stress environments pose additional threats for polymer-encapsulated actives such as carotenoids. Under H_2_O_2_ and HOCl, the main product is apo-13-carotenone-5,6-epoxide (minor: β-apo-12′-carotenone-5,6-epoxide), with first-order kinetics in carotene and second-order in H_2_O_2_, evidencing rapid polyene scission. Free carotene retained only 63% after 120 min in 8.82 M H_2_O_2_ and 34% after 30 min in 15.18 mM HOCl, whereas chitosan–tripolyphosphate complexes maintained >94% stability for 300 min against both oxidants (encapsulation efficiency 92.70%; loading 2.631%; recovery 87.99%), preserved hydroxyl-radical scavenging (slightly reduced reducing power), and released by diffusion to 88.33% cumulative over nine days. The cationic polyelectrolyte network thus offsets extracellular oxidative degradation that would otherwise compromise targeted delivery [21].

The immunological dimension of PLGA acidification extends to innate cells: degradation of PLGA nanoparticles reduced medium pH from 7.28 to ≈6.4 by week 3 (mass loss 0 to 18.93%; Mn 5.46 ± 1.33 to 1.36 ± 0.30 kDa; Mw 13.30 ± 1.44 to 3.84 ± 1.20 kDa; PDI 2.29–3.04). THP-1-derived macrophage viability remained high at day 2 for pH 7.28–6.7 but fell to <50% at pH 6.4 by day 4; Annexin V positivity rose to ~50% at pH 6.4 while remaining <5% at pH 7.28–7.0. Cells up-regulated M1 markers (CCR7) and secreted TNF-α and IL-1β proportionally to acidification, identifying pH ≤ 6.5 as a critical threshold for viability/apoptosis and quantifying acid-driven immunostimulation, supporting buffering or slower autocatalysis in PLGA designs [22].

At the systems level, biodistribution of PLGA carriers is non-linear, with rapid early degradation (~30%) preceding slowed clearance and preferential sequestration in liver, bone marrow, lymph nodes, spleen, and peritoneal macrophages. Release is typically biphasic: an initial burst from surface-associated drug followed by an erosion–diffusion phase governed by autocatalytic hydrolysis, both highly dependent on composition and geometry. Among compositions, 50:50 PLGA degrades fastest versus 65:35, 75:25, and 85:15; higher Mw slows degradation; higher surface-area-to-volume ratios accelerate mass loss; alkaline or strongly acidic media increase hydrolysis; and internal carboxyl end-groups autocatalyze breakdown near neutrality. Targeting-relevant liabilities include dose dumping at high loads, inconsistent release from evolving crystallinity, Tg drift, porosity changes, and interaction-driven switches between bulk and surface erosion. Numerically anchored guidance follows ~30% early degradation, composition ranking with 50:50 most labile, shorter release goals favoring amorphous, higher-hydrophilicity matrices, and six-month regimens requiring higher Mw or semi-crystalline constructs, always balanced against microenvironmental acidity and immune activation [23].

Oral site-selective polymer platforms add manufacturing and anatomical constraints. Coating adhesion differs across gelatin vs. HPMC shells; solvent-based processes risk API instability; non-uniform coating thickness drives dissolution variability. Quantified targeting includes Enprotect^®^ HPMC/HPMC-AS releasing 38% in jejunum, 50% in ileum, and 12% in cecum with ~40 min post-gastric onset across prandial states; Nefecon^®^ coated capsules opened in the distal ileum in 10/12 volunteers (Peyer’s patch targeting); Mycapssa^®^ maintained biochemical control in acromegaly with 85.7% of patients at IGF-1 < 1.3 × ULN while avoiding injection-site AEs. Material choices set disintegration windows (PVA-PEG/HPC ≈ 60 min; HPMC-AS ≈ 420 min; EC non-disintegrating in rats), underscoring mismatch risk between intended and actual release. Practical issues include solvent ingress into fills, coating damage during filling from diameter growth, and the need for seal coats to buffer moisture/oxidation, each critical for polymeric oral targeting systems aiming at GI site selectivity [24].

**Figure 10:**
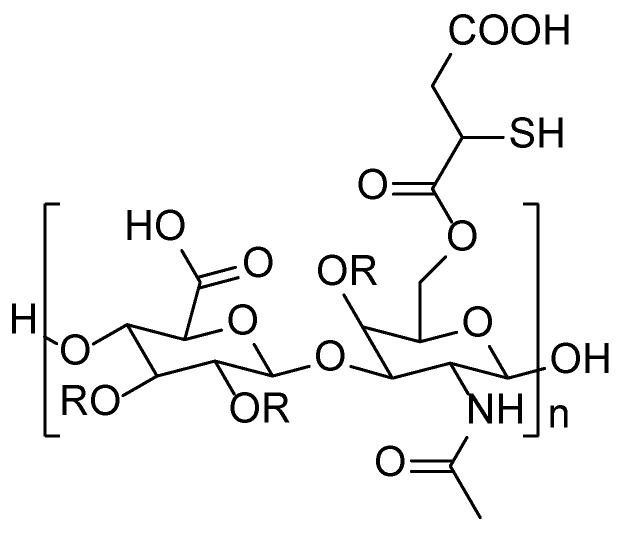
Structure of HAMS.

Finally, enzyme-responsive polysaccharides can address nonspecific degradation and release. A thiolated hyaluronic acid excipient (HAMS) (Figure 10), formed by esterifying HA with mercaptosuccinic acid (degree of thiolation 10.03 ± 3.6%; 250.18 ± 90.32 µmol g^−1^ thiols; pKa 3.2, 4.2, 8.8; solubility 50.99 ± 0.02 g L^−1^), irreversibly inhibits vertebrate hyaluronidase (Hyal) while remaining a substrate for bacterial hyaluronate lyase (Hysa), thereby switching release to pathogen microenvironments. Ionotropic-gelated nanoparticles with an enzyme-responsive M23-polyphosphate core and HAMS coating (265 ± 47 nm; ζ ≈ −25 mV) preserved colloidal integrity for 7 days at 37 °C, whereas HA-coated controls agglomerated. Alkaline phosphatase triggered only ≲0.3 nm phosphate from M23-PP/HAMS over 144 h (vs. ~0.4 nm from M23-PP/HA and ~400 nm from uncoated M23-PP in 24 h), confirming suppression of off-target release; Michaelis–Menten analysis showed BT-Hyal lower Vmax and Km on HAMS than on HA (higher affinity, inhibited turnover). Functionally, M23-PP/HAMS achieved 8-log killing of *S. aureus* ATCC 25923 in a race-for-the-surface co-culture without osteoblast toxicity, shifting to bacteriostasis (98% reduction) when Hysa was inhibited, indicating Hysa-triggered release. The core M23-PP exhibited 39 < MIC ≤ 156 nM and MAC = 39 nM, unchanged by HAMS. HAMS thus emerges as the most active polymer in this study for selective pathogen-triggered release while mitigating mammalian enzyme-driven degradation and dose dumping in targeted anti-infective delivery [25].

### 1.4. PEG’s Limitations in Overcoming Biological Barriers

Across delivery platforms, the dominant sources of failure arise not from carrier design but from the body’s own transport architecture: steep filtration bottlenecks, competitive protein sequestration, and spatially fragmented perfusion networks restrict how much of a circulating dose can traverse biological barriers and enter target tissues. Meta-analysis employing physiologically based pharmacokinetic (PBPK) modeling across 376 datasets collected between 2005 and 2018 has revealed that the central limitation in nanoparticle-mediated drug targeting lies in the intrinsically low tumor delivery efficiency. The mean and median percentages of the injected dose (%ID) at the final sampling time were reported as 2.23% and 0.76%, respectively, with temporal dependence demonstrating a decline from 2.24% and 0.76% at 24 h to 1.23% and 0.35% at 168 h following intravenous administration, indicating progressive loss from tumor tissue over one week. The PBPK model successfully reproduced 83% of tumor kinetic profiles (R^2^ ≥ 0.75). Sensitivity analysis identified the tumor distribution coefficient (PT), endothelial permeability to tumor cells (PATC), the maximum tumor cell uptake rate (Kmax, T), and the tumor volume fraction (VTC) as major determinants of delivery efficiency. Subgroup analyses further revealed modest gains associated with specific design features: active targeting yielded a median of 0.89% ID compared with 0.70% ID for passive targeting; inorganic nanomaterials outperformed organic ones (1.12% ID versus 0.62% ID), with dendrimers (7.96% ID) and iron oxide nanoparticles (2.80% ID) as respective top performers. Rod-shaped geometries achieved 1.62% ID versus 0.74% ID for spherical particles. Hydrodynamic diameters below 10 nm (1.41% ID) and above 200 nm (0.94% ID) performed marginally better than intermediate sizes (0.56–0.75% ID), while near-neutral or positive ζ-potentials (0.81–0.90% ID) surpassed negative ones (0.47% ID). Notably, no improvement in delivery efficiency was observed after 2015 (mean 2.33% ID versus 2.13% ID before 2015), and statistical analyses confirmed that low tumor delivery strongly correlates with diminished distribution and permeability coefficients (*p* < 0.01), emphasizing mechanistic barriers in extravasation and intratumoral transport. Collectively, these findings delineate the nanoparticle delivery problem as one rooted in limited extravasation, retention, and cellular internalization under heterogeneous enhanced permeability and retention (EPR) conditions, with only minor improvements achievable through ligand-mediated targeting or variations in composition and morphology [26].

Complementary reanalysis based on classical pharmacokinetic (PK) parameters has demonstrated that the interpretation of nanoparticle tumor delivery is highly dependent on the chosen metric. The frequently cited median of approximately 0.7% ID in tumors underrepresents actual relative delivery by nearly two orders of magnitude when compared with exposure-based ratios. Evaluation of 136 matched tumor–blood datasets from 117 publications revealed a weak correlation between %ID and the ratio of the area under the curve (AUC_tumor_/AUC_blood_). Although the overall median tumor accumulation was 0.67% ID, the median exposure ratio reached 76.12%, suggesting that tumor exposure typically ranges from half to equal that of blood exposure, rather than representing a minute fraction of the administered dose. Subset analyses further indicated median values of 0.55% ID (45.46% AUC ratio) for liposomes, 0.68% ID (143.94% AUC ratio) for polymeric nanoparticles, and 0.64% ID (81.44% AUC ratio) for inorganic particles. Importantly, %ID correlated only moderately with tumor Cmax and poorly with exposure-based metrics, highlighting a methodological pitfall in using %ID as the sole measure of delivery efficiency. The study thus recommends the use of validated PK endpoints, particularly AUC ratios, for accurate cross-comparison between targeted polymer systems and small-molecule controls in vivo [27].

A broad quantitative assessment of nanoparticle tumor delivery across 232 datasets from 117 studies conducted between 2005 and 2015 further confirmed the low delivery ceiling of polymeric and related nanocarriers. The median tumor accumulation was 0.7% ID, showing no improvement over the decade, meaning that only about seven of every 1000 administered nanoparticles reach the tumor, while approximately 99% remain off-target. This inefficiency imposes significant challenges in terms of cost, scaling, toxicity, and feasible dosing volumes. Multivariate analysis identified tumor model and cancer type as primary predictors of delivery, with material composition and physicochemical parameters contributing secondarily. Inorganic particles exhibited slightly higher delivery (0.8% ID) than organic polymeric systems (0.6% ID), and sub-100 nm sizes (0.7% ID) modestly outperformed larger ones (0.6% ID). Near-neutral surface charge (−10 to +10 mV) corresponded to 0.7% ID, outperforming both positive (0.6% ID) and negative (0.5% ID) surfaces. Active targeting achieved 0.9% ID versus 0.6% ID for passive delivery, and rod-shaped geometries demonstrated higher accumulation (1.1% ID) compared with spheres (0.7% ID) and plates (0.6% ID). Only four studies reported delivery exceeding 5% ID, all characterized by near-neutral charge and particle size under 100 nm. Theoretical dose projections at 1.0% ID underscore the impracticality of achieving therapeutic concentrations in humans, requiring ~2.7 × 1014 drug-encapsulated or ~6.4 × 1014 surface-loaded particles per patient, corresponding to injection volumes of ~90 mL and ~213 mL, respectively, at 5 nM colloid concentration. The analysis attributes the low delivery primarily to rapid clearance via the mononuclear phagocyte system (MPS) and renal filtration, as well as to restricted extravasation through abnormal tumor vasculature and extracellular matrix, establishing a quantitative upper limit that current polymeric nanocarriers must overcome [28].

Mechanistic analysis further elucidates the systemic, microenvironmental, and cellular barriers underlying these delivery inefficiencies. During circulation, nanoparticles undergo opsonization and protein corona formation, leading to macrophage uptake and size-dependent clearance, renal filtration prevailing below ~10 nm, while complement activation and rapid MPS removal dominate above ~200 nm. Surface charge exerts a major influence on circulation time, with neutral to slightly negative carriers showing the longest systemic persistence, whereas cationic and highly anionic formulations are rapidly cleared. Hemodynamic shear and vascular flow hinder nanoparticle margination and adhesion, though non-spherical geometries such as rods and ellipsoids exhibit improved vessel wall interactions, consistent with their higher tumor accumulation (~1.1% ID). Immunological barriers add further complications: PEGylated systems, although designed for stealth, can elicit anti-PEG antibody responses leading to accelerated clearance and, in rare cases, anaphylaxis. Intracellular barriers also limit efficacy due to poor endosomal escape, necessitating the inclusion of ionizable or stimuli-responsive components. Moreover, polymeric nanocarriers exhibit class-specific drawbacks, low encapsulation efficiency in lipid–polymer hybrids, predominant hepatic and splenic uptake, and aggregation or cytotoxicity unless cross-linked or otherwise stabilized. Despite several FDA-approved nanomedicines, polymer-based systems remain underrepresented, reflecting translational barriers driven by patient heterogeneity and suboptimal physicochemical designs. Collectively, these findings define the major challenges for polymeric nanomedicine delivery as sub-percent tumor accumulation, extensive off-target sequestration, size and charge-dependent clearance trade-offs, and protein corona–mediated loss of specificity, with only near-neutral, rod-shaped, ~100 nm actively targeted carriers showing marginally superior performance yet still constrained by endosomal and organ-level distribution limitations [29].

Quantitative in vivo imaging of ligand-coated, PEG-backfilled nanoparticles further clarifies the cell-type-specific distribution barrier. For trastuzumab- and folic-acid-targeted gold and silica nanoparticles, whole-tumor delivery efficiencies were 0.59% ID and 0.25% ID, respectively, compared with PEG-only controls. Critically, only 0.001–0.003% ID interacted directly with SKOV-3 cancer cells, while tumor-associated macrophages (TAMs) captured 0.020–0.038% ID, representing 13.26% and 12.48% of TAM populations versus only 0.96% and 0.42% of cancer cells being nanoparticle-positive at 24 h. Each TAM internalized 1.6–2.1× more particles than tumor cells, and extracellular matrix sequestration accounted for 88.2–99.9% of intratumoral particles. Although targeting reduced hepatic burden from 62.33% to 31.30% ID, TAM-dominated uptake persisted across materials and sizes, including 15 nm, 55 nm, and 100 nm AuNPs and 100–140 nm silica nanoparticles. Spatial analyses revealed that 70.4% of tumor vessels were surrounded by perivascular TAMs, decreasing from 37.7% within 10 µm to 14.3% at 50 µm distance, indicating macrophage-mediated shielding. Matrigel transport assays demonstrated exponential decay in nanoparticle diffusion, with less than 8% of cells reachable within 24 h, and macrophage–cancer co-cultures confirmed competitive uptake (47.1% versus 11.4%). Cytotoxicity assays with trastuzumab–doxorubicin–AuNPs showed macrophage-preferential killing (34.3% macrophage death vs. 0% cancer-cell death at high doses), suggesting that apparent therapeutic effects may derive from stromal cell engagement rather than direct cancer targeting. Altogether, these results reveal that dominant TAM capture, extracellular matrix sequestration, and diffusion limits constitute the principal barriers to effective polymer-stabilized nanoparticle delivery, with only minor benefits from ligand functionalization even in optimized 55 nm gold systems [30].

Modern research continues to expand the landscape of polymeric carriers, exploring both natural and synthetic systems with diverse and often dual biological functions. Among these, bentonite-based materials [31], chitosan [32], gelatin [33], and a wide array of engineered polymers [34,35] have emerged as compelling candidates, each offering distinct structural and physicochemical advantages. Bentonite-derived polymer clay hybrids are receiving increasing attention due to the clay’s natural abundance, biocompatibility, and well-characterized interlayer chemistry [36] that enables tunable loading and release behaviors. Yet, while such platforms reflect the field’s creative breadth, the most promising polymer classes driving transformative advances in drug delivery, namely polyoxazolines (POx), polyglycerols (PG), zwitterionic polymers, polypeptides, and polypeptoids, are discussed in detail in the following sections. Extensive evaluation in preclinical and, in some cases, clinical studies enables informed assessment of their safety, immunogenicity, and feasibility, enabling assessment of their translational potential.

## 2. Poly(2-oxazoline)s (POx)

**Figure 11:**
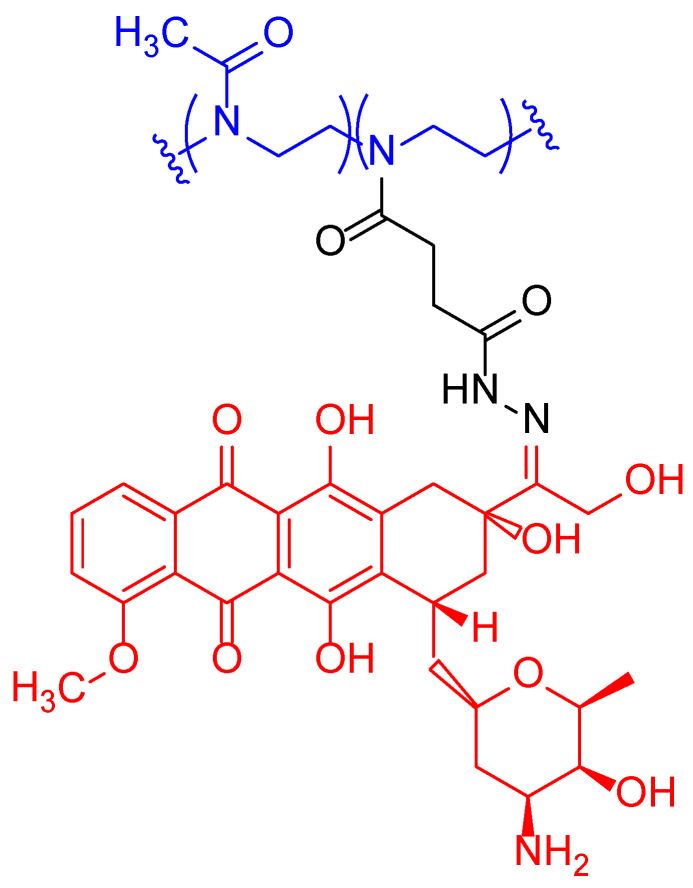
Structure of PMeOx-Dox.

Acid-labile poly(2-oxazoline) (POx)–anthracycline conjugates exemplify how corona chemistry and size tuning can couple lysosomal unmasking with pharmacokinetic control. PMeOx–doxorubicin (MD1–MD4) (Figure 11) and PEtOx–doxorubicin (ED1–ED4) were synthesized using hydrazone linkers to trigger release at endo-lysosomal pH, with hydrodynamic diameters positioned near the renal threshold: ~7.5–7.8 nm for ~30 kDa constructs to favor rapid clearance and ~9–10 nm for ~45 kDa constructs to bias toward EPR-mediated tumor deposition. Drug contents were MD1 7.1 wt%, MD2 8.1 wt%, MD3 12.6 wt%, MD4 15.2 wt%; ED1 8.0 wt%, ED2 8.4 wt%, ED3 10.7 wt%, whereas ED4 15.6 wt% became insoluble in PBS at 37 °C, marking a formulation limit at very high loading. Doxorubicin liberation was markedly faster at pH ~5 than at pH 7.4, consistent with lysosomal unmasking; uptake in B16 and SKOV-3 cells proceeded predominantly via clathrin-mediated endocytosis, with PMeOx carriers outperforming PEtOx in antifouling and cellular uptake. The most active construct was PMeOx–DOX MD4 (15.2 wt%, ~9.5 nm), while the PEtOx analog at matched loading suffered aqueous insolubility, a practical drawback for delivery [37].

For mucosal targeting, methacrylated PEOZ derivatives created by partial hydrolysis and subsequent methacrylation yielded thermo-responsive, mucoadhesive polymers with a clear performance optimum. Cloud points (Tcp) increased with methacrylation, MA10PEOZ 70 °C, MA25PEOZ 74 °C, MA35PEOZ 79 °C, before over-hydrophobization reduced solubility for MA55PEOZ at 37 °C. On ex vivo sheep nasal mucosa, methacrylation-dependent retention after a 5 min wash was ~35% (MA10PEOZ), 42% (MA25PEOZ), and ~50% (MA35PEOZ), with MA35PEOZ surpassing a cationic glycol chitosan control (*p* < 0.05); unmodified PEOZ showed no retention. Irritation and cytotoxicity markers remained comparable to PBS controls (slug mucus production 2–3%, no HEK293 viability loss across soluble grades), defining ~35% methacrylation as a favorable safety–adhesion window for nasal residence extension [38].

Beyond single-function excipients, POx architectures enable stimuli-responsive targeting and on-demand release. Thermo-responsive micellization arises from side-chain hydrophobicity tuning; PMeOx remains hydrophilic without an LCST in water, whereas increasing hydrophobicity (e.g., toward PBuOx) introduces LCST behavior. POx–based assemblies and hydrogels exploit temperature-induced shrink–swell transitions to trigger payload liberation. An anti-fouling PEtOx/PAA multilayer achieved thickness control from ~12 nm to 1.5 µm, stabilized by thermal crosslinking (150 °C, 40 min) to prevent dissolution above pH = 4.5 while retaining pH-responsive swelling; a light-addressable copolymer raised LCST by ~15 °C upon 350 nm irradiation, enabling optical modulation of phase behavior. POx functionalization of polydopamine-coated black phosphorus nanosheets enabled dual delivery of doxorubicin and bortezomib with tumor-pH charge reversal (~6.8), NIR-assisted uptake/photothermal action, and pH-accelerated release; in vivo imaging indicated predominant intratumoral doxorubicin localization. Collectively, these data identify doxorubicin and bortezomib as key active payloads and quantify architectural handles (12 nm–1.5 µm multilayers; 150 °C/40 min crosslinking; ~15 °C LCST shift) that directly modulate targeted delivery [39].

POx grafting can also tune biopolymer transport through mucus. Gellan gum grafted with short PEtOx chains (living PEtOx Mn ≈ 7960 g/mol) reduced mucoadhesion and promoted mucus penetration for ocular/transmucosal use. Process optimization established 50 °C as the reaction temperature to balance reactivity and gellan integrity (70 °C degraded the backbone; ≤40 °C promoted unfavorable gelation). DOSY NMR confirmed covalent attachment and intramolecular GG–PEtOx contacts, yielding a more compact macromolecular conformation while flagging partial backbone degradation as a formulation risk. L929 cytocompatibility (0.01–1 mg/mL) met regulatory requirements (≥85% viability at 2, 4, 7 days) with significant proliferation between time points and no concentration-dependent toxicity. Functionally, GG forms transparent, ion-triggered gels in tear fluid for prolonged precorneal residence, while PEtOx grafts are intended to minimize mucoadhesion to aid penetration, together positioning GG-g-PEtOx as a biocompatible transmucosal carrier pending payload validation [40].

**Figure 12:**
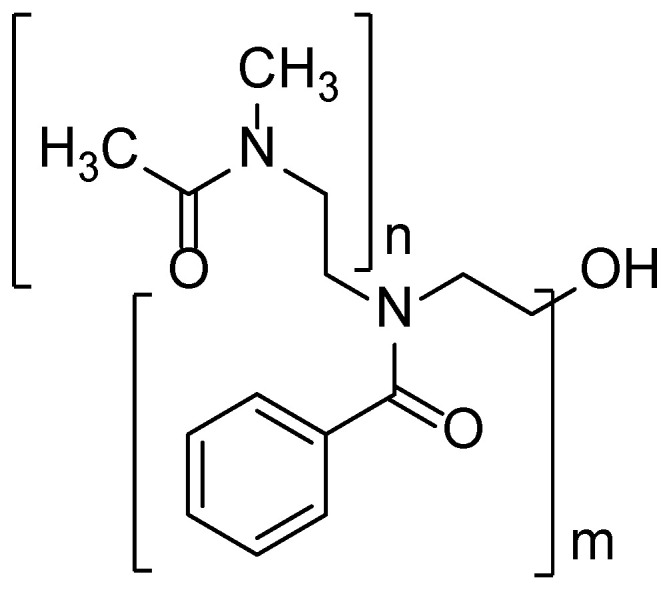
Structure of PMeOxz-grad-PPhOxz polymer.

**Figure 13:**
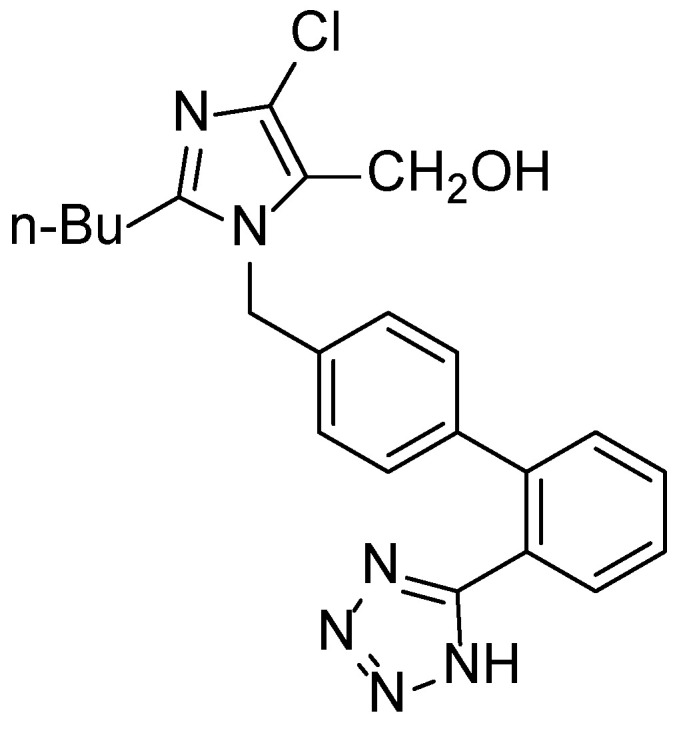
Structure of losartan.

Nanostructure–payload interplay is equally evident in gradient POx carriers for small molecules. An amphiphilic gradient PMeOxz72-grad-PPhOxz28 (Figure 12) (Ɖ 1.14; Mn ≈ 4900–5200 g/mol) formed unimers (Rh ~3–4 nm), micelles (~13 nm), and aggregates (~96–150 nm; ζ ≈ +4 mV). Losartan (Figure 13) loading at 20 wt% yielded micelles (Rh ~12 nm) and ~117 nm aggregates; at 50 wt% loading, micelles shrank (~10 nm), and aggregates decreased (~59–60 nm) with increased scattering and ζ trending toward neutrality (+2 to +1 mV). NOESY NMR resolved specific π–π and alkyl–aryl contacts between losartan and PPhOxz/PMeOxz segments; DOSY and ATR-FTIR corroborated tight inclusion. Serum-background DLS showed colloidal stability with multimodal nanometric populations, and 5 h ultrasound dialysis indicated strong drug retention. The 50 wt% system was the most active formulation owing to tighter packing, smaller aggregates, higher mass loading, and near-neutral ζ—features favorable for in vivo compatibility and prolonged retention [41].

As localized transmucosal matrices, chitosan/PEtOx blend films (CHI/POZ) incorporating 0.1% *w*/*v* ciprofloxacin offered manufacturability and efficacy. All films were uniform (0.05–0.08 mm thick), flexible (folding endurance > 300 for CHI and 80:20 CHI/POZ; 65 ± 5 for 40:60), and exhibited surface pH 3.76–4.14, appropriate for vaginal application. Drug-loaded films showed reduced optical transmittance yet enhanced release: 8 h dissolution reached 42 ± 2% (CHI) versus 56 ± 1% (CHI/POZ 40:60). Antimicrobial testing revealed weak intrinsic activity for drug-free blends against *E. coli* (zones 13.3 ± 2.1–21.0 ± 2.0 mm) and none for CHI alone, while drug-loaded films were broadly active and outperformed ciprofloxacin disks, with CHI/POZ 40:60 most active against *S. aureus* (46.1 ± 1.7 mm) and CHI/POZ 60:40 against *E. coli* (42.5 ± 2.2 mm). All films were mucoadhesive, with adhesion decreasing as POZ content increased, supporting POZ as a functional oxazoline excipient that boosts release and antimicrobial efficacy while maintaining acidic surface pH and biocompatibility [42].

Stealth performance of POx shells in blood is critical for systemic targeting. Core-cross-linked micelles with POx/POzi shells showed minimal leukocyte association in fresh human whole blood when the shell was PMeOx: <10% Cy5-positive across granulocytes, monocytes, B, NK, and T cells, markedly lower than a polycationic PDMAEMA control that caused 51.3% hemolysis at 100 µg/mL (8.0% at 10 µg/mL). The association rank was PMeOx < PEtOx < PMeOz < PEtOz; removing plasma generally reduced interactions. Within PEtOx variants, the degree of polymerization modulated association (PEtOx100 < PEtOx75 < PEtOx50), and plasma pre-incubation increased B-cell association for PEtOx50/75 but not PEtOx100. Drug-free sizes and dispersities were: PMeOx75 49 ± 1 nm (PDI 0.09), no LCST; PEtOx50 37 ± 1 nm (PDI 0.41), Tcp 66 °C; PEtOx75 41 ± 1 nm (PDI 0.19), Tcp 63 °C; PEtOx100 34 ± 0 nm (PDI 0.09), Tcp 64 °C; PMeOz75 36 ± 1 nm (PDI 0.18), no LCST; PEtOz75 134 ± 3 nm (PDI 0.21), Tcp 27 °C. Thus, PMeOx provided the most stealth across six leukocyte subsets, with PEtOx100 the least interacting within its series [43].

For polynucleotide delivery, amine-bearing gradient POx copolymers demonstrate the value of secondary amines. Three chemotypes, POxN1pendant (primary amines), POxN2pendant (secondary amines in side chains), and POxN2main (secondary amines in main chain), self-assembled at 7.5 mg/mL into structures of D_h_ 153 nm, 67 nm, and 69 nm, respectively. Upon DNA complexation (N:P 3, 7, 10), secondary-amine systems formed progressively smaller polyplexes: DNA–POxN2pendant from 911 nm (N:P 3) to 120 nm (N:P 10) and DNA–POxN2main from 710 to 140 nm, whereas POxN1pendant yielded mixed populations up to 300 nm plus >1 µm aggregates and displayed a Tcp drop from 62 to 27 °C at N:P 3. Bare DNA ζ was −140 mV; only POxN1pendant complexes reached positive ζ (consistent with cationic character), whereas POxN2 systems formed small-positive/near-neutral ζ polyplexes, milder than PEI controls. POxN2pendant emerged as the most active DNA carrier, producing compact nanoparticles at N:P 7–10 with acceptable cytocompatibility in HT-1080 cells and confirmed transfection (secreted luciferase) across a defined viability–efficacy window [44].

**Figure 14:**
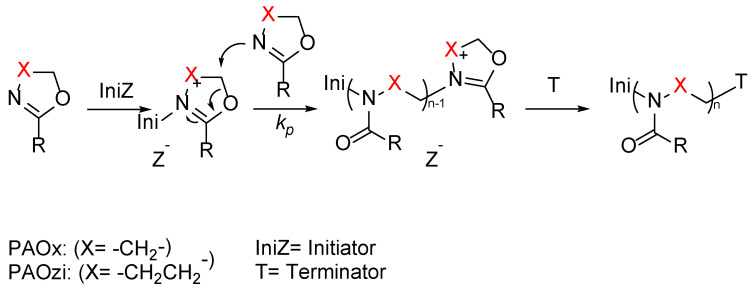
Mechanism of the CROP of cyclic imino−ether monomers.

Architecturally, arm-first core-cross-linked star POx unify high drug loading with lysosome-relevant release triggers. Hydrophilic PMeOx arms (99% conversion in 7 min; 90% isolated yield) were grown by microwave-assisted cationic ring-opening polymerizations (CROP) (Figure 14) and cross-linked into hydrophobic P(PhBisOx-cl/co-ButOx) cores (97% isolated yield; star conversion 83–99%). SEC-MALS showed Mw, star 3.73 × 10^5^–8.96 × 10^5^ g/mol with ~44–106 arms (Mn, arm ≈ 8.2–8.3 kDa). The unimolecular micelles efficiently encapsulated doxorubicin and released faster at pH = 5.2 than 7.1; blanks were cytocompatible in HeLa, while DOX-loaded stars elicited concentration-dependent cytotoxicity. The most robust carriers combined PMeOx arms with a PhBisOx core diluted by ButOx to enhance free volume and encapsulation [45].

Dual pH/temperature-responsive PAOx blocks extend control over assembly and delivery. Post-polymerization amidation of methyl-ester-functionalized PAOx installed tertiary amines to afford PC3DIPEDOx150 and PMeOx-b-PC3DIPEDOx (20/130; 40/110; 60/90) with Ɖ ≤ 1.27 and Mn 27.4–38.6 kDa. Quantitative functionalization was verified by ^1^H NMR (loss of 3.58 ppm methyl ester; appearance of 0.93 ppm diisopropyl signals). LCST behavior was pH-dependent: at pH = 9, only PC3DIPEDOx150 showed a Tcp (12.1 °C); at pH 10, all displayed LCSTs (15, 14, 13, 10 °C, respectively). For PMeOx60-b-PC3DIPEDOx90 at pH 10, DLS revealed a z-average jump from 50 nm (4 °C) to >2000 nm near 10 °C, fragmentation to ~130 nm at 14 °C, and stabilization at ~55 nm (PDI 0.15), defining a heat-triggered nanoparticle window with a PMeOx stealth corona. In DPBS/DMEM, thermoresponse shifted, yet preserved nanoparticle formation. These tertiary-amine PAOx micelles co-loaded Rhodamine B-C18 and paclitaxel, suppressing MDA-MB-231 proliferation; PMeOx60-b-PC3DIPEDOx90 was the most active carrier by combining low Tcp at mildly basic pH, stable nanoparticles, narrow PDI, and a low-fouling corona [46].

Thermoresponsive stomatocytes from POx–PLA diblocks illustrate mesoscale gating for hyperthermia-assisted delivery. PEtOx20-b-PDLLA120 initially formed ~600 nm vesicles (PDI 0.09) that deflated to ~200 nm upon dialysis (75 mM NaCl, 5 °C), with cryo-TEM membrane thickness ~23 nm. Blending PEtOx20-b-PDLLA120 with PiPrOx64-b-PDLLA119 (4:1 *w*/*w*; fPEtOx ≈ 10%) yielded ~200 nm stomatocytes whose necks narrowed and irreversibly closed on heating to ~40–42 °C, exploiting the PiPrOx LCST (~43 °C); in contrast, PEtOx20-b-PDLLA83 or PEtOx30-b-PDLLA130 predominantly produced smaller micelles (15–40 nm) and 300–450 nm polymersomes that lacked sufficient membrane area/solvent plasticization for stomatocyte formation. The 4:1 PEtOx:PiPrOx blend was thus the most functionally active for hyperthermia-triggered gating near physiological temperatures [47].

**Figure 15:**
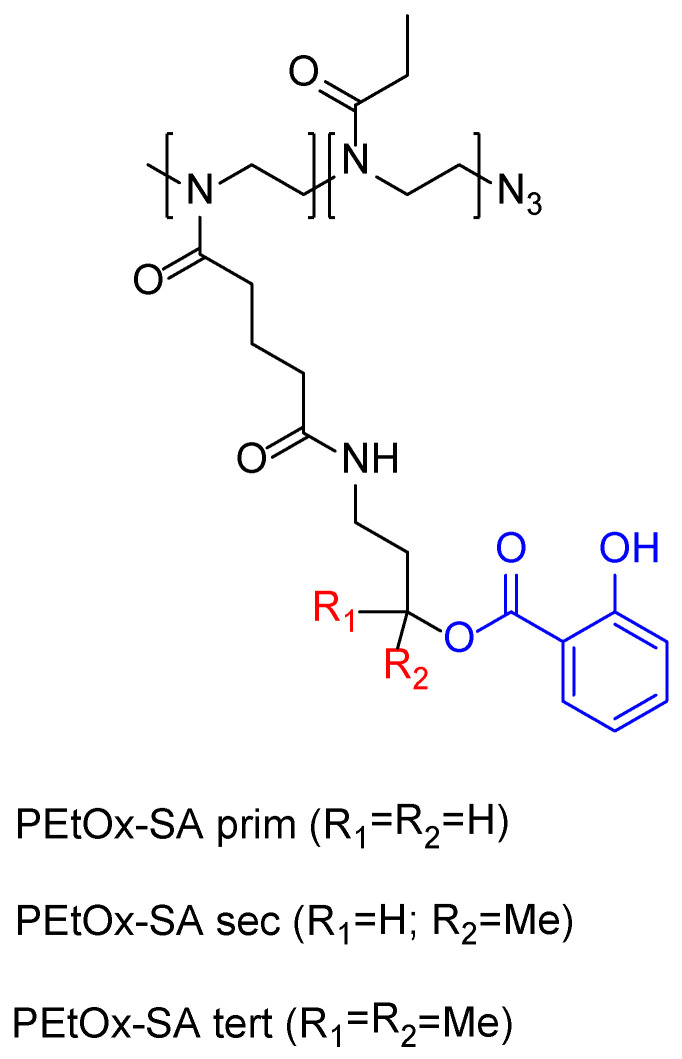
PetOx−SA derivatives structure.

As covalent prodrugs, side-chain-hydroxylated PEtOx–salicylic acid conjugates (Figure 15) (PEtOx-SAprim and PEtOx-SAsec; Mn ≈ 11.5 kDa; Ɖ 1.21) provided ester-linkage-programmed kinetics. SA loadings were 6.7% (primary-alcohol) and 4.2% (secondary-alcohol). At 37 °C, <1% SA was released after 24 h at pH 1 (unless heated to 70 °C); at pH 8.5, PEtOx-SAprim released ~80% in 24 h (complete by ~48 h), whereas PEtOx-SAsec released ~40% in 24 h (complete by ~4 days). In PBS pH 7.4, both exhibited slow, month-long sustained release without burst, with PEtOx-SAprim approximately twice as fast as PEtOx-SAsec; esterase modestly accelerated PEtOx-SAprim and minimally affected PEtOx-SAsec, indicating steric shielding. PEtOx-SAprim thus emerged as the more active construct for base-triggered delivery and tunable long-acting release in neutral media [48].

**Figure 16:**
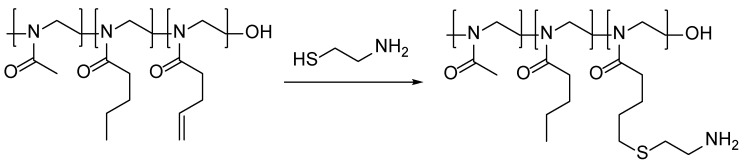
Synthesis route of nucleobase−crosslinked POx nanoparticles.

**Figure 17:**
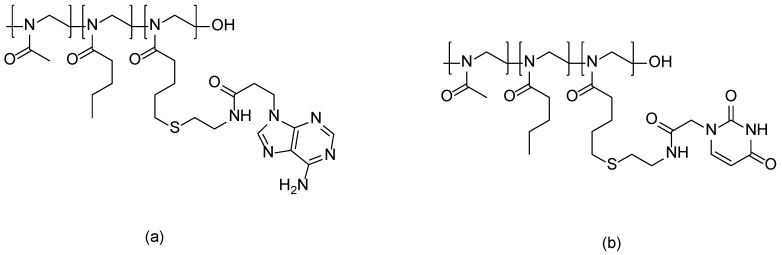
Structures of (**a**) POxA and (**b**) POxU.

Core stabilization addresses the common problem of micelle disassembly in blood. Nucleobase-crosslinked POx nanoparticles, assembled from adenine- and uracil-functionalized amphiphiles at 1:1 (Figure 16), generated smaller, more stable carriers with ultrahigh paclitaxel loading (38.2% *w*/*w*), prolonged circulation, enhanced tumor accumulation, and deeper penetration relative to uncross-linked POx controls. The synthesis provided high yields (A-COOEt 86.1%, A-COOH 91.0%, U-COOH 81.6%, POxA 85.1%, POxU 87.2%) (Figure 17). Addressing a known liability, ~80% dissociation of PEG-PCL/PEG-PDLLA micelles to unimers under serum shear followed by Kupffer-cell clearance, and short half-life (2.8 h) of a prior POx PTX micelle (similar to Taxol, 2.6 h), the nucleobase-reinforced cores improved antitumor efficacy in E0771 and 4T1 models at 10 mg/kg PTX and increased tumor PTX levels at 4–24 h versus non-crosslinked POx. Paclitaxel was the most active payload, with adenine–uracil crosslinks the key stabilizing motif [49].

**Figure 18:**
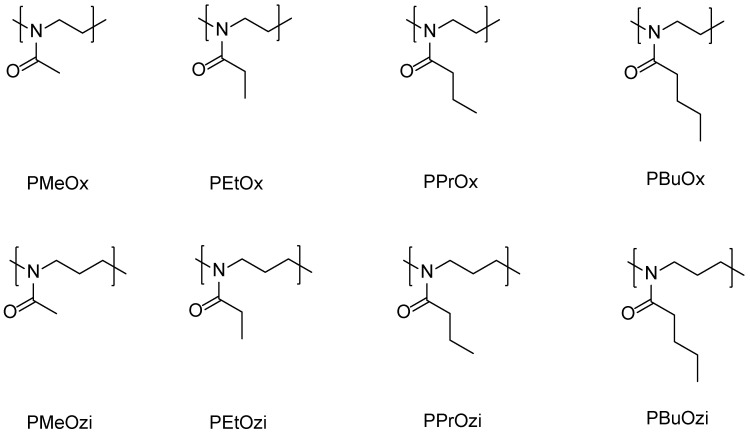
Structures of different coronas and cores.

A broader consolidation highlights the targeting profile of PAOx/PAOzi. Hydrophilic coronas (PMeOx, PEtOx, PMeOzi) and hydrophobic cores (PBuOx, PAOzi) (Figure 18) support high-capacity micelles; PEtOx was non-cytotoxic up to 80 mg/mL at 3 h (moderate effects ≥ 20 mg/mL at 12–24 h), and PMeOx was non-cytotoxic up to 40 mg/mL at 12 h. In vivo, PAOx micelles exhibited a maximal tolerated dose of ~500 mg/kg in mice, and repeated i.v. dosing of PEtOx up to 2 g/kg in rats caused no adverse effects. Protein resistance was strong (PMeOzi brushes outperforming PMeOx and PEG), complement activation generally low-to-moderate (small C3a-desArg increase; negligible iC3B), and immunogenicity mixed (reduced virus-like particle recognition; no anti-PEG binding; yet IgM and ABC reported for PMeOx-liposomes in rats, underscoring formulation dependence). Radiotracer studies placed the renal filtration threshold for PEtOx near 40–50 kDa and showed longer plasma residence and slightly higher 24–78 h tumor uptake for 40 kDa PEtOx–doxorubicin than equal-mass PHPMA–doxorubicin; a PEtOx–rotigotine prodrug has entered Phase 1. Design guidance emphasizes tunable LCST/hydrophobicity, wormlike vs. spherical micelles with reduced opsonization, and near-neutral ζ and sub-100 nm sizes to balance EPR capture with post-therapy renal elimination; leading cargos include paclitaxel, doxorubicin, docetaxel, and etoposide [50].

Manufacturable transmucosal dosage forms based on PEtOx demonstrate simultaneous improvements in dissolution and bioadhesion for poorly soluble drugs. A mucoadhesive buccal film produced by hot-melt extrusion with 20% *w*/*w* fenofibrate showed that blending 200 kDa PEtOx with 20% polyethylene oxide (F8) achieved >95% drug release in 2 h (>5-fold solubility enhancement vs. free drug) and increased mucoadhesion 2.2–2.7-fold (peak force and work) relative to controls. Mechanical properties were tunable via HPC/HPMC/PEO; F8 exhibited EB% 161.1 ± 14.9 and TS 8.9 ± 1.7 MPa. All films maintained mucosa-compatible surface pH (6.47–7.10), thickness 0.88–1.21 mm, USP content uniformity, and 1-month stability at 25 °C/60% RH (f_2_ > 50). PEtOx–PEO F8 thus emerged as the most active oxazoline-based matrix for mucoadhesive transmucosal targeting by coupling rapid, complete release with enhanced adhesion and robust manufacturability [51].

Kierstead et al. [52] conducted a comparison of polymer-modified liposomes, clarifying the paradoxical behavior of poly(2-methyl-2-oxazoline) (PMOX) in vivo. When evaluated after a single intravenous administration, PMOX–lipid conjugates (2.1–2.5 kDa, Đ ≈ 1.04–1.09) incorporated at 5 mol% into approximately 100 nm liposomes exhibited outstanding stealth properties, yielding a circulation half-life of 30.5 ± 2.5 h and an AUC of approximately 2600 in rats, values essentially identical to PEG and substantially higher than those of HPMA, PVP, PDMA, PAcM, or unmodified liposomes. This prolonged circulation was accompanied by reduced mononuclear phagocyte system uptake, with <20% of the injected dose recovered in the liver and <10% in the spleen at 48 h.

This initial pharmacokinetic advantage was not sustained upon repeated administration. Following a low priming dose of 0.1 μmol phospholipid/kg and a second injection seven days later, PMOX-modified liposomes underwent a pronounced accelerated blood clearance response, with the circulation half-life collapsing to ~1.9 ± 0.5 h and the AUC falling to ~89, corresponding to a >95% reduction in systemic exposure and a marked increase in hepatic and splenic accumulation. In contrast, liposomes modified with HPMA, PVP, PDMA, or PAcM maintained comparable pharmacokinetic profiles across doses and did not exhibit ABC.

Analysis of the underlying mechanism indicated that repeated dosing failure resulted from potent polymer-specific IgM-mediated immune recognition. Anti-PMOX IgM levels increased sharply from baseline (~0.12 ± 0.01) to 2.58 ± 0.50 post-dose, with a mean reciprocal endpoint titer of 2094, exceeding that observed for PEG (316), while no cross-reactivity between anti-PEG and anti-PMOX IgM was detected.

Poly(2-oxazoline)s feature a chemically stable tertiary amide backbone, unlike the oxidation-prone ethers in PEG. Synthesized from N-substituted 2-oxazoline monomers, their side chains can be tuned–short, hydrophilic groups like ethyl or methyl promote solubility and stealth. The amide groups form strong hydrogen bonds with water, creating a dense hydration shell that blocks protein adsorption and immune recognition. Lacking charged or aromatic motifs, POx avoids triggering innate sensors or antibody formation. Flexible, neutral, and cleanly hydrated, POx moves invisibly through the immune landscape.

## 3. Polyglycerols (PGs)

Polyglycerol (PG) scaffolds have emerged as versatile, low-fouling platforms to address long-standing liabilities in targeted delivery by combining receptor selectivity, stimulus-responsive release, and high payload compatibility within biocompatible architectures. In macrophage-centric antiparasitic therapy, mannose-decorated dendritic PG carriers (PG-PEG-Mann) bearing 5, 10, or 20 mannose units and coupled to amphotericin B (AmB) via a pH-sensitive linker achieved selective uptake into Leishmania infantum-infected macrophages, with the five-mannose construct maximizing internalization (~30% in human and up to 88% in murine macrophages). Entry proceeded by phagocytosis/pinocytosis (~80%) and clathrin-mediated endocytosis (~79%), confocal microscopy confirmed co-localization with intracellular parasites, and AmB release was efficient at pH 4.0 while stable at pH 7.4. Representative compositions for PG–AmB–PEG–Mann5 featured AmB loading 0.43 wt%, mannose 0.41 wt%, and optional FITC 0.29 wt%, collectively demonstrating macrophage tropism with environment-triggered release and reduced off-target deposition [53].

Extending PG utility to the skin, hyperbranched PG-functionalized β-cyclodextrins (hPG-βCD) were engineered as macromolecular crosslinkers within pNIPAM nanogels to enhance dermal delivery of hydrophobes. These βCD-tNGs elevated Nile Red (NR) solubility to ~120 μg mL^−1^ and increased ex vivo porcine-skin penetration to 3.33 μg cm^−2^, outperforming free NR, β-CD, and unmodified gels. Crosslinker acrylation tuned nanogel rigidity/hydrophilicity, with 5k-6A and 8k-6A yielding the optimal loading–penetration balance; cloud points of 32–37 °C enabled release under physiological skin temperatures, and microscopy corroborated deeper NR distribution associated with stratum corneum hydration [54].

In the investigation conducted by Yuanwei Pan et al. [55] at the tumor–stem-cell interface, dendritic PG-gated mesoporous silica (mSiO2-dPG) nanoparticles (~88.4 ± 1.2 nm; 588.2 m^2^/g^−1^; 0.84 cm^3^/g^−1^) co-delivered doxorubicin (DOX) and the P-gp inhibitor tariquidar (Tar) via acid-cleavable Schiff bases and CD44-targeting hyaluronic acid. In 3D mammospheres, mSiO_2_-dPG(DOX, Tar)-HA enhanced nuclear DOX accumulation and reduced CSC viability by >60% at 48 h (5 µg/mL^−1^ DOX equivalent), suppressed SOX2/OCT4/NANOG (>70%), diminished tumorsphere formation (>50%), depleted ALDH+ subpopulations, and induced caspase-3 activation—together overcoming CSC-associated multidrug resistance.

To coordinate intracellular release with enzymatic cues, esterase-responsive dendritic PG nanogels (dPG-NGs) were synthesized by iEDDA click crosslinking (dPG-tetrazine × dPG-norbornene), forming ~146 nm (cryo-TEM) colloids that encapsulated avapritinib (BLU-285) and 17-AAG at theoretical 5 wt% loads with near-quantitative efficiency for single-drug systems (DLC 4.95 wt%). Exposure to CALB accelerated size reduction to ~20 nm by day 6 (vs. day 11 in PBS), and serum stability (189–215 nm, near −2 mV) with <5% hemolysis demonstrated compatibility. In GIST-T1 models harboring D842V ± G680R mutations, dual-loaded NGs restored sensitivity beyond single agents, confirming efficient intracellular co-delivery and release [56].

Polyglycerol can also stabilize 2D photothermal chemotherapeutic platforms: PG-functionalized MoS2 nanosheets (~80 nm), further modified with folate and lipoic acid (FP-MoS2), achieved extraordinary DOX and chloroquine loadings (88.9 wt% and 92.4 wt%) with pH-sensitive release (DOX 28.3% and CQ 31.4% at pH 5.5/40 h; markedly lower at pH 7.4). Near-infrared irradiation (808 nm, 1 W cm^−2^) raised the temperature by ~45 °C (3 min, 200 μg/mL^−1^), accelerating release. In DOX-resistant HeLa-R cells, dual delivery plus NIR reduced survival to 18.3% at 48 h; spheroid studies showed deeper DOX penetration and nuclear localization via folate targeting, while PG functionalization improved biocompatibility (>90% viability at 160 μg/mL^−1^) [57].

As PEG-sparing stealth lipids in LNPs, linear PG (lPG) amphiphiles (e.g., DMG-lPG and block variants) matched PEG controls in mRNA encapsulation/transfection while abrogating anti-PEG recognition. lPG-LNPs displayed diameters ~223–264 nm (vs. 110 nm PEG-LNPs), high encapsulation (lPG 93.4% vs. PEG 98.3%), near-neutral ζ (−3.30 to −6.78 mV), bilayer shells with dense cores by cryo-TEM, and stability over 3–4 weeks at 4 °C. Anti-PEG IgG binding was negligible (0.50–0.83 ng/mL^−1^) compared with PEG-LNPs (816–1310 ng/mL^−1^). HepG2 viability exceeded 80% (up to 95.4% for lPG-block-MeOlPG), and eGFP mRNA transfection reached ~43% with MFIs 3068–3890, including activity at 0.05 μg/mL^−1^—substantiating lPG as a next-generation stealth lipid [3].

**Figure 19:**
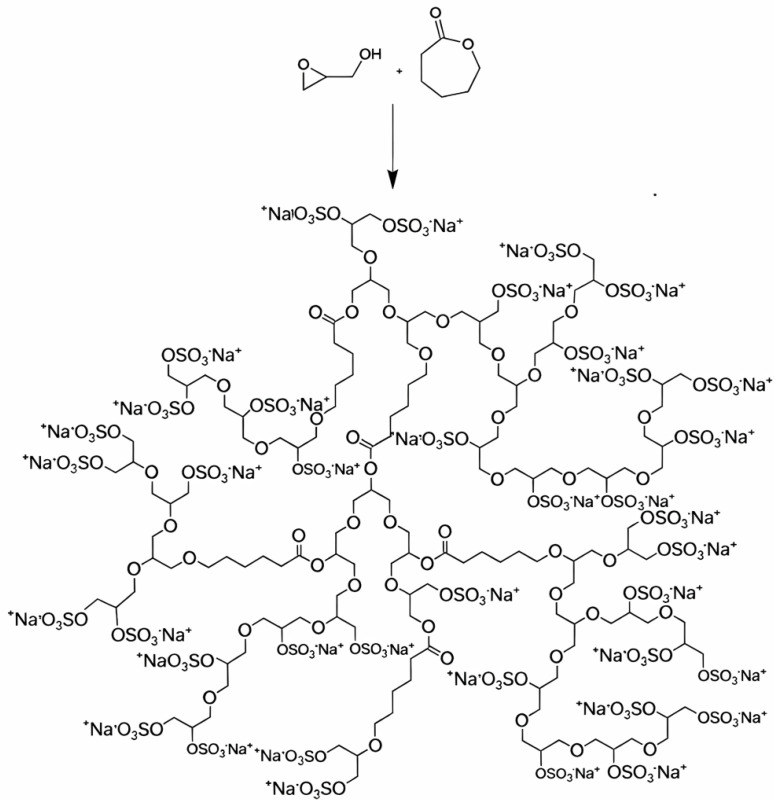
Synthesis of dPGS−PCL.

Bridging scale-up and efficacy, dendritic PG sulfate–poly(caprolactone) (dPGS-PCL; ~40–60 kDa) (Figure 19) produced on a 20 g scale loaded sunitinib (~5 wt%) and exhibited controlled, milieu-dependent release (n = 0.83 at pH 7.4; 0.63 at pH 5.8; 0.72 with elastase). Cytotoxicity improved over free drug (A431 IC_50_ 2.86 μg/mL^−1^; HT-29 7.16 μg/mL^−1^ vs. 9.19 μg/mL^−1^), with very low carrier toxicity (IC_50_ ≥ 2077 μg/mL^−1^). In vivo, dPGS-PCL@sunitinib accumulated in HT-29 tumors within 24 h and, at 10 mg/kg^−1^ i.p., matched the tumor inhibition of 30 mg/kg^−1^ oral free drug, indicating enhanced bioavailability and reduced dosing burden [58].

Thermoresponsive two-dimensional hyperbranched PG interlocked with pNIPAM (2D-PNPG; lateral 200–500 nm; thickness 20–50 nm) afforded high antibiotic loads (amoxicillin 70 wt%; tetracycline 80 wt%) with LCST-gated release at pH 7.4. AMX exhibited temperature-dependent efficacy (efficient *S. aureus* inhibition at 37 °C but not 27 °C), whereas TC released steadily and inhibited *E. coli* at both temperatures (~40% release at room temperature sufficed), demonstrating selective antibacterial performance through thermal gating and drug–matrix interactions [59].

**Figure 20:**
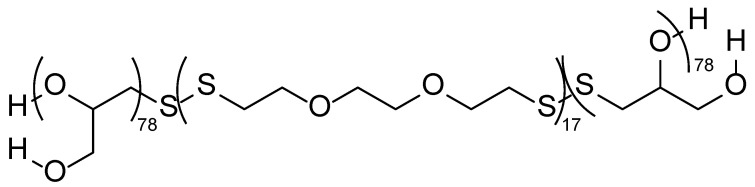
Structure of PG−b−polydisulfide−b−PG block.

Chemically cross-linkable PG also underpins redox-responsive vesicles: amphiphilic PG-b-polydisulfide-b-PG (Figure 20) triblocks self-assembled into ~230 nm polymersomes (CAC 0.02 mg mL^−1^) with unusually high doxorubicin loading (DLE 54%; DLC 16%). Intracellular-level glutathione (1–20 mM) triggered rapid disassembly and DOX release; carriers were hemocompatible and cytocompatible (>90% viability to 1.0 mg/mL^−1^ polymer), while DOX-loaded vesicles produced time- and dose-dependent cytotoxicity (48 h killing > 50% at ~4.0 μg/mL^−1^ DOX in HeLa; ~8.7 μg/mL^−1^ in MDA-MB-231). Elevated cellular GSH accelerated killing, confirming disulfide exchange as the central mechanism [60].

Head-to-head comparisons with PEG across PG topologies underscore intrinsic antifouling and delivery advantages. Linear, hyperbranched, and dendritic PGs (degree of branching 0.53–0.62; Mw 6–870 kDa) showed negligible cytotoxicity and <1% hemolysis, reduced fibrinogen/albumin adsorption on gold/glass by >96% at 4 h and >90% at 24 h, and suppressed bacterial adhesion by >99%, exceeding PEG coatings. In vivo, hybrid linear–branched PG conjugates prolonged circulation beyond PEG analogues with minimal organ accumulation. As carriers, PG derivatives achieved >80% encapsulation for hydrophobes (e.g., Nile Red, DOX), tunable cloud points (10–90 °C), and improved liposome stability versus PEGylated vesicles, supporting sustained payload retention [61].

These antifouling benefits extend to ultrathin device coatings: plasma-activated grafting of hyperbranched PG (0.3–1.3 nm) onto titanium, silicon, PTFE, nitinol, and stainless steel yielded uniform, pinhole-free layers (C–O–C 67.6–83.8% of O environments) that reduced albumin/fibrinogen adsorption below detection after rigorous washing, well under the ~30 ng/cm^−2^ fibrinogen threshold for platelet activation. HPG uniquely prevented irreversible protein adsorption versus ethanol plasma polymer controls, decreased bacterial adhesion by >3 logs, and blocked biofilm maturation. Ex vivo whole-blood assays showed minimal platelet adhesion/activation; in ApoE^−^/^−^ mice, HPG-coated stents maintained 100% patency at 28 days without neutrophil/complement activation or local inflammation [62].

As functional biointerfaces, succinic-acid–functionalized dPG (19 kDa; ~81 acids per macromolecule) coated ~20 nm immunomagnetic nanoparticles for selective EpCAM-positive CTC capture (MCF-7 93.6%; SKBR3 90.04%; A549 67.3%; HeLa 2.6%), retaining performance in serum (82.7%) and whole blood (76.6%). Captured cells were embedded in thiol–acrylate hydrogels (dPG-acrylate × 4-arm PEG-thiol, 3–5% *w*/*v*) that supported spheroids > 300 μm and yielded dose-dependent responses to doxorubicin/paclitaxel more predictive than 2D assays, aided by the antifouling dPG layer that minimized nonspecific blood interactions [63].

**Figure 21:**
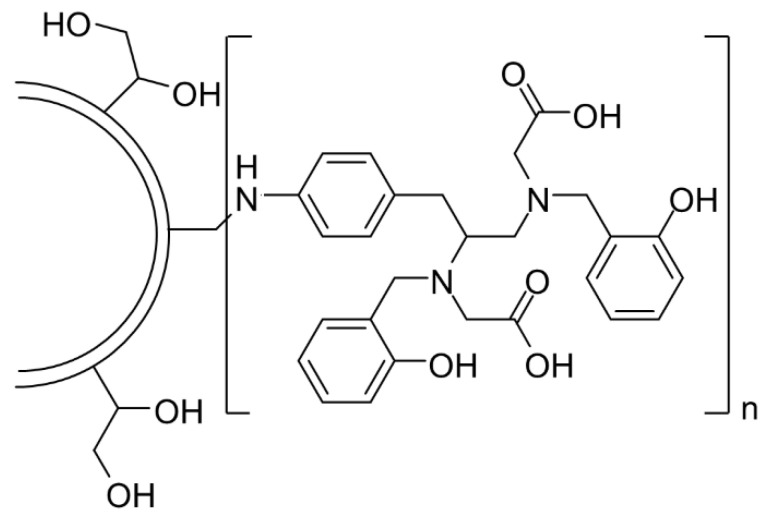
HPG−HBEDS conjugates.

Beyond delivery, PG scaffolds can serve as multivalent macrochelators: HPG-HBED conjugates (Figure 21) (100/200 kDa; 25–244 chelators per polymer) were highly soluble (≤12.1 mM in PBS, pH 7.4) and bacteriostatic against *S. aureus* (up to 80% inhibition at ~2.4 mM), while limited against *P. aeruginosa*. As adjuvants, the highest-valency conjugates combined with rifampicin (0.0075 μg/mL^−1^) produced 100% *S. aureus* inhibition and improved MRSA suppression (~25% over rifampicin alone); no synergy occurred with ciprofloxacin, gentamicin, or penicillin G, and antagonism was observed in some pairs. Cytocompatibility (~80% fibroblasts; ~70% HeLa at 2 mM) contrasted with free HBED toxicity [64].

Finally, PGylation of biologics can match PEGylation’s pharmacokinetic benefits while maintaining activity. Site-selective N-terminal conjugates of anakinra with linear PG (5–40 kDa) or hyperbranched PG showed size-consistent hydrodynamic radii (Ana-20-hPG more compact at 8.02 nm vs. Ana-20-LPG 10.53 nm; Ana-20-PEG 10.99 nm), preserved secondary structure with ~2 °C Tm gain, and superior proteolytic protection for larger LPGs (~60% intact at 2.5 h for ≥10 kDa LPG vs. ~34–36% for native/Ana-5). Binding to IL-1R1 remained low-nanomolar (native KD 0.02 nM; Ana-10-/20-/40-LPG 0.16/0.26/0.33 nM; Ana-40-PEG 0.25 nM). In mice, terminal half-life extended from 2.3 h (native) to 10.1 h (Ana-40-LPG) and 11.1 h (Ana-40-PEG), with AUC increases (1.8 to 12.9/17.0 nmol·h·mL^−1^) and reduced clearance (100.0 to 17.4/16.5 mL·h^−1^·kg^−1^), establishing PG as a viable, structurally tunable alternative to PEG for half-life extension [65].

Safety profiling for pulmonary use delineates concentration limits for polyglycerol fatty-acid esters (PGFAs). PG3-C16/C18 partial ester (HLB 5.1; mp 56.5 °C) and PG2-C18 full ester (HLB 2.6; mp 59.4 °C), alone or mixed, were well tolerated by A549 epithelium and J774A.1 macrophages up to 1 mg/mL^−1^, but higher exposures induced lysosomal accumulation, reduced phagocytosis (less than 50% at 10 mg/mL^−1^), increased nitric oxide/cytokine release, and phospholipidosis; ALI epithelium preserved TEER ≤ 1 mg/mL^−1^. High-pressure-homogenized microsuspensions (d50 2–6 μm; ζ −20 to −30 mV) were stable, with partial esters less toxic than full esters and mixtures intermediate—defining a ≤1 mg/mL^−1^ biocompatibility window for lung formulations [66].

Numerous researchers have made it evident: the molecular configuration of PG directly governs its biocompatibility and ability to evade immune detection. Polyglycerol, especially in its hyperbranched form, builds a soft, water-rich shield through dense hydroxyl groups. This fully hydrophilic layer blocks protein binding and keeps immune sensors at bay. To preserve this stealth, synthesis must avoid carboxylic acids, amines, and aromatic fragments, as any charged or reactive moieties could trigger recognition. PG stays invisible because it gives the immune system nothing to hold on to.

## 4. Zwitterionic Polymers

Zwitterionic polymers have matured into a distinct class of stealth and targeting materials that leverage dense hydration shells and ultralow protein adsorption to address long-standing liabilities of PEGylated systems while enabling high drug loading and microenvironment-responsive release. Recent syntheses on biodegradable backbones—polylactide, polycaprolactone, polyphosphoesters, polycarbonates, polyacetals, peptides, and polysaccharides—demonstrate protein adsorption suppressed below 5 ng/cm^−2^ (vs. 10^−20^ ng/cm^−2^ for PEG), 2–3-fold longer circulation half-lives than PEGylated analogues, and sustained antitumor performance with drug loading contents of 10–30 wt% and encapsulation efficiencies of 70–90%. In xenograft models, zwitterionic drug conjugates (e.g., p(EK-co-C-SPA-ss-Dox)) achieved >75% tumor inhibition compared with ~55% for Doxil, extended plasma half-life beyond 20 h (PEG ~8 h), and maintained >90% enzymatic activity for protein cargos after systemic passage, collectively establishing zwitterionic platforms as biodegradable, low-immunogenic alternatives that preserve efficacy with reduced systemic toxicity [4].

Within this framework, albumin-mimetic zwitterionic polypeptides have enabled synchronized pharmacokinetics and intratumoral activation. Poly(glutamatyl lysine-co-cysteine) carriers bearing sulfo groups and doxorubicin (Dox) via hydrazone linkers combined PEG-like half-life with pH-triggered uptake: at pH 6.7, internalization into MCF-7 cells approached free Dox and exceeded Doxil, whereas macrophage uptake at pH 7.4 fell to 27.4 ± 7.6% of Doxil. Biodistribution shifted toward the tumor over the liver/kidney prior to half-life, and 18-day therapy yielded 93.2 ± 3.0% tumor inhibition versus 54.2 ± 6.5% for Doxil with higher body mass retention, attributing superior efficacy to the synergy of long circulation and rapid, acidic-pH internalization [67].

Zwitterionic shells can be integrated with orthogonal triggers to orchestrate on-demand drug release. A hypoxia-sensitive vehicle, DHigh-PEI-(A + P), grafted with the zwitterion DMAAPS (*N*,*N*-dimethyl(acrylamidopropyl) ammonium propane sulfonate), an AZO hypoxia-cleavable linker, and the photosensitizer PpIX, coassembled paclitaxel (>35% loading) into antifouling nanoparticles (less than 0.3 ng cm^−2^ protein adsorption) that circulate and accumulate in 4T1 tumors. Light-activated PpIX aggravated and homogenized intratumoral hypoxia, accelerating AZO cleavage and disassembly with robust PTX release, while serum stability minimized premature leakage. In vitro and in vivo, photodynamic preconditioning amplified cytotoxicity and tumor suppression with minimal collateral toxicity, illustrating how zwitterionic antifouling can be coupled to hypoxia engineering for precise spatiotemporal delivery [68].

Charge-conversion strategies further exploit the tumor’s acidic milieu. Zwitterionic-to-cationic polyprodrug nanomedicines (ZTC-NMs) combined a poly(carboxybetaine)-like corona with a GSH-responsive camptothecin prodrug to form ~50 nm particles that remained neutral at pH 7.4 but switched to >+20 mV within 3 h at pH 6.6, boosting membrane interactions and uptake. CPT loading reached 14.9%, and GSH (1^−10^ mM) liberated >50% free CPT within 48 h (LC–MS confirmed). PET and biodistribution in A549 models showed sustained circulation yet higher, longer tumor retention for ZTC-NMs than nonresponsive zwitterions; therapeutically, ZTC-NMs (3 mg/kg^−1^ CPT-eq, q3d × 5) maximized tumor control and survival without weight loss typical of free CPT [69].

**Figure 22:**
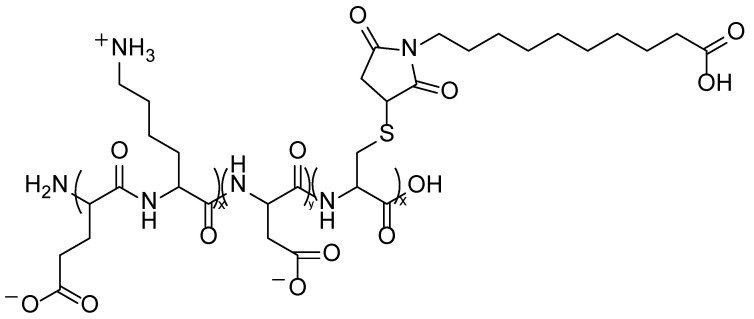
EKDCM micelles structure.

Albumin-mimicking negative-biased zwitterionic polypeptides provide an additional route to high-capacity, metabolizable nanodrugs. EKDCM micelles (E/K/D/C with MUDA-cross-linked cores) (Figure 22) maintained ζ ≈ −8 to −12 mV after Dox loading, achieved stable ~64–84 nm sizes (TEM-confirmed), and delivered 15–16 wt% Dox at ~85% efficiency without aggregation in 10% FBS over 4 days. Release was accelerated at pH 5.5–6.7 (50% in 8–12 h) and by trypsin digestion, correlating with a β-strand/unordered to α-helix transition that facilitated drug liberation; in MCF-7 cells, nuclear accumulation and dose-dependent cytotoxicity matched or exceeded free Dox, aligning high loading, serum stability, and enzymatic degradability with rapid intracellular release under tumor conditions [70].

Beyond oncology, zwitterionic carriers can self-target acidic infectious niches. Comparative analyses show full ζ-potential reversal under biofilm-like acidity for select systems (e.g., PQAE within 2 h; ZTC-NM ~3 h), enabling in vivo accumulation in staphylococcal biofilms where conventional liposomes failed. Effective isoelectric points clustered around pH 6.2–7.0 (e.g., DCPA-H_2_O, ZTC, PQAE), whereas classic sulfobetaines/phosphobetaines with pI ≈ 2–3 lacked charge-reversal capacity. Although prolonged circulation and minimal protein adsorption favor targeting, acidity’s lack of disease specificity motivates dual-responsive designs that integrate hypoxia, ROS, or enzymatic triggers to sharpen selectivity [71].

Zwitterionic polymer–drug conjugates can multiplex therapies while preserving stealth. A sulfobetaine-modified PLA conjugate (30.1 wt% SB) bearing paclitaxel (6.5 wt%) and gemcitabine (17.7 wt%) formed ~45.6 nm nanoparticles (ζ ≈ −5 mV) with 1.8-fold higher cellular uptake than free dye in MIA PaCa-2 cells and superior cytotoxicity above 0.1/0.24 μg mL^−1^ (PTX/GEM). In vivo, 74.5% signal persisted at 1 h and 18.7% at 24 h with diminished hepatic/renal sequestration; treatment significantly inhibited tumors with increased cleaved caspase-3, reduced Ki67, and clean blood chemistry, underscoring the biocompatibility of sulfobetaine-based conjugates [72].

Hybrid inorganic–organic nanocomposites also benefit from zwitterionic shells. Superparamagnetic SPIONs (~9.5 ± 1.9 nm cores) coated with PMPC-b-CLnDopa produced 160–190 nm colloids that retained magnetothermal functionality, sustained dexamethasone release from the zwitterionic layer, and exhibited high biocompatibility (HeLa viability indistinguishable from controls at 0.05–0.2 mg/mL^−1^) with efficient uptake; size-tuned variants (e.g., RhB-labeled MP730) showed enhanced cytoplasmic accumulation, highlighting catechol anchoring and zwitterionic stabilization as complementary handles for tumor-directed delivery [73].

Biodegradable zwitterionic matrices can couple antifouling with acid-triggered payload liberation. SBMA-grafted PLA/PEAd copolymers (Mn 3.2–4.9 kDa) formed paclitaxel nanoparticles (220–565 nm) with water contact angles ~78° after SBMA grafting, hemocompatibility by hemolysis tests, and strong pH-dependent mass loss (8–83% over 30 days) and release (up to ~79% at pH 5.0 vs. ~18–35% at pH 7.4), achieving >80% MDA-MB-231 killing while sparing iMEFs, particularly in PEAd-rich formulations that degraded faster under acidic conditions [74].

**Figure 23:**
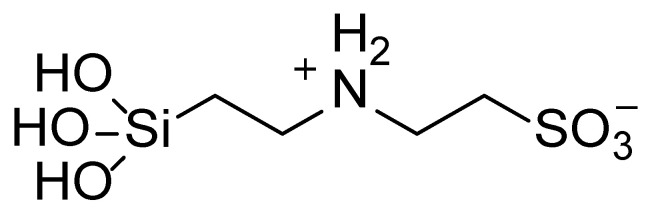
Sulfobetaine-grafted silica (SiZwit) polymer structure.

Material-agnostic insights into zwitterionic hydration layers also translate to silica. Sulfobetaine-grafted silica (SiZwit) (Figure 23) expanded hydrodynamic size to 174–185 nm due to a two-layer water shell (bound water 40.1% in DI; 36.7% in PBS), remained charge-neutral at pH 7–9 with maximal hydration at pH 8.5, and improved colloidal stability > four-fold while cutting protein adsorption (BSA/FBS) by up to 90%. Macrophage uptake fell by 86–93% versus amine-silica, yet loading of zwitterionic levofloxacin increased 3.7-fold with sustained release to 96 h and preserved antibacterial activity after washing, validating simultaneous antifouling, reduced clearance, and high payload retention [75].

Polyurethane prodrug micelles provide another zwitterionic route to endo/lysosomal activation. A DHCB-based carboxybetaine polyurethane covalently linked to Dox (PU-hyd-DOX) self-assembled into ~100 nm micelles stable at pH 7.4 (20% release/50 h) yet releasing >80% at pH = 5 within 35 h. The carrier matrices were nontoxic (>90% viability), and PU-hyd-DOX outperformed PEG-PLGA@DOX against HepG2 cells with higher uptake within 3–5 h, consistent with zwitterion-enabled pH-triggered protonation and enhanced affinity for negatively charged tumor membranes [76].

An interesting feature is that zwitterionic blocks can be tailored for the oral, parasite-targeted delivery of hydrophobes. Amphiphilic PBMA-SBMA and PBMA-MESBMA formed ~100 nm nanoparticles and ~20 nm micelles, respectively, with the latter showing superior aqueous stability and selective binding to parasitized RBCs (74.8% targeting; ≤0.8% healthy RBCs), rising to 82.6% after curcumin encapsulation. Drug loading reached 0.3 mg^−1^ polymer (PBMA-MESBMA), in vitro antiplasmodial potency matched free curcumin (IC_50_ ~5 μM), and oral dosing in *P. yoelii* models elevated 1 h plasma concentrations 8.9-fold over free drug, with confocal imaging confirming intra-parasite entry across erythrocytic stages and negligible hemolysis up to high doses, establishing zwitterionic micelles as selective, orally viable vectors for antimalarials [77].

Zwitterionic polymers carry tightly paired positive and negative charges within each monomer, creating a net-neutral, superhydrophilic shell through strong ion–dipole interactions. To sustain this stealth, the opposing charges must remain close, within the same monomer unit, avoiding local imbalances that attract plasma proteins. Hydrophobic or asymmetric elements break this harmony and should be excluded during synthesis.

## 5. Polypeptides and Peptoids

Protein- and polypeptide-based nanocarriers have emerged as intrinsically biodegradable, bioactive, and biocompatible scaffolds that support targeted and stimuli-responsive delivery, with albumin nanoparticles representing a clinically validated archetype. Abraxane (albumin-bound paclitaxel) exemplifies reduced systemic toxicity with enhanced tumor accumulation, while vanillin-crosslinked albumin carriers achieved doxorubicin (Dox) IC_50_ = 3.69 μg/mL^−1^ versus 4.00 μg/mL^−1^ for free Dox via pH = 6.5 and 20 mM glutathione–dependent release and showed lower cardiac deposition. Additional albumin formats improved antitumor efficacy (gemcitabine-albumin in A549 xenografts) and preserved antileukemic activity with reduced endothelial permeability (dasatinib-albumin). Beyond albumin, biomimetic HDL particles transporting simvastatin or tanshinone IIA reduced cholesterol, triglyceride, and LDL with discoidal HDL localizing to plaques and driving dose-dependent regression; simvastatin rHDL extended half-life and attenuated inflammatory signaling, and HDL–gold hybrids enhanced SR-B1-mediated tumor imaging/theranostics while diminishing lymphoma growth upon repeat dosing. Gelatin systems likewise afforded pharmacokinetic and pharmacodynamic gains: type-A gelatin improved indomethacin exposure and anti-edema effects; gelatin–PEG–noscapine reduced MCF-7 IC_50_ and increased plasma recovery; ultrasound-responsive gelatin–tPA restored thrombolysis; and cationized gelatin plasmid complexes enabled efficient ocular transfection. Casein nanoparticles extended flutamide half-life, zein carriers increased curcumin bioavailability and cellular uptake, and zein–tocopherol–PEG–daidzin conjugates raised plasma AUC 2.4-fold. Hydrophobin–gold composites encapsulating paclitaxel reduced IC_50_ by ≈100-fold versus free drug, remained stable in vivo for four days, and disassembled in response to intracellular glutathione, collectively underscoring albumin, gelatin, zein, and hydrophobin derivatives as versatile platforms across oncology, cardiovascular, and inflammatory indications [78].

**Scheme 1:**
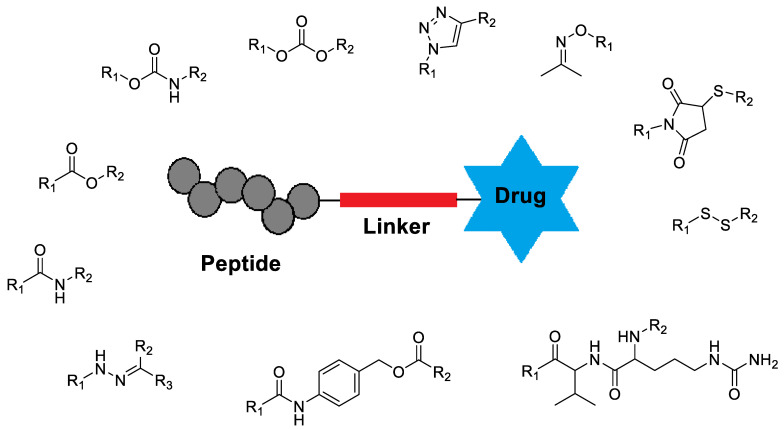
Peptide–drug conjugate with common linkers.

Extending this paradigm, peptide–drug conjugates (PDCs) leverage linker chemistry (Scheme 1) to program intracellular liberation and pharmacokinetics. Ester/amide linkers in a gemcitabine–[D-Lys6]–GnRH conjugate produced tumor inhibition at 18.8 μmol/kg^−1^ (vs. 454.5 μmol/kg^−1^ for free drug) with sustained exposure despite partial ester hydrolysis, while an MMP-2-activatable MAHNP–doxorubicin hybrid for HER2+ tumors reduced volume by 74.7 ± 5.1% (free Dox 53.4 ± 6.6%) and extended t_1/2_ to 17.6 h (vs. 7.7 h). Peptide 18-4-Dox conjugates highlighted the potency cost of stable amide-amide bonds (IC_50_ 18–19 μM) versus ester–amide designs (0.9–1.5 μM) while preserving cancer selectivity and activity against Dox-resistant lines. ANG1005 (Angiopep-2-paclitaxel × 3 via succinyl linkers) crossed the BBB via LRP-1, increased brain uptake 4.5-fold, lowered IC_50_ 2–3×, improved survival in glioblastoma and lung-metastasis models, and advanced to phase II. Clinically, 177Lu-DOTA-TATE established PDC radiotherapy via somatostatin receptor 2 targeting. Carbamate linkers (e.g., JF-10-81) stabilized camptothecin delivery to SSTR2 + tumors, suppressing growth in CA20948 and NCI-H69 while sparing SSTR2-negative PC-3, together demonstrating that ester, amide, carbamate, disulfide, and hydrazone chemistries tune 70–80% tumor inhibition, multi-fold stability enhancement, and receptor-specific delivery with minimal off-site toxicity [79].

To achieve sustained release of bioactive peptides, elastin-like polypeptide (ELP) depots with redox-responsive crosslinks have been engineered as injectable hydrogels. Recombinant cELPs (ELP[A14V1C1-160] and ELP[V15C1-160]) formed disulfide-stabilized networks within ≈3 min at 4 °C and maintained integrity for up to three weeks at 20–37 °C with gradual elastase degradation. Enfuvirtide (T^−20^) loaded into 5 wt% cELP2 hydrogels exhibited diffusion-controlled release sustaining antiviral levels for 168 h with EC50 comparable to free peptide and minimal cytotoxicity (CC_50_ > 100 μM). In rats, subcutaneous cELP2 depots (3.3 wt%, 9–18 mg/kg^−1^ T^−20^) extended plasma exposure to 8 h (free T^−20^ t_1/2_ ≈ 2 h) while blunting C_max_, with residual gel confirming ongoing release and benign local histology at day 10, supporting reduced injection frequency and peak-related adverse effects for antiviral polypeptide therapy [80].

Concurrently, polypeptidic shuttles and PDCs are redefining noninvasive CNS delivery, where <2% of small lipophiles and virtually no biologics traverse the BBB unaided. Receptor-mediated or adsorptive-mediated transcytosis with ≤50-residue shuttles, identified by phage panning (e.g., pepC7, LRPep2) or derived from endogenous ligands (Angiopep-2/D-Angiopep-2, ApoE/ApoB fragments), increases endothelial transport by >10- to 100-fold, raising CNS drug levels 3–5-fold while halving systemic exposure. ANG1005 exemplifies this strategy with 4.5-fold higher brain uptake and >70% tumor inhibition in intracranial models. Cationic viral-origin CPPs (TAT, RVG29) further enable neuron-selective delivery of siRNA and chemotherapeutics with >80% gene silencing in murine brain. Collectively, charge distribution, secondary structure, and receptor selectivity allow tuning of pharmacokinetics and off-target profiles, establishing polypeptides and polypeptoid-derived BBB shuttles as precision vectors for CNS therapeutics [81].

Reflecting the importance of trans-tissue penetration, the cyclic 9-mer iRGD integrates an RGD motif for αvβ3/αvβ5 binding with a cryptic CendR sequence that, upon proteolysis, engages neuropilin-1 to trigger bulk transport into tumor parenchyma. With nanomolar integrin affinity and dual receptor engagement, iRGD enhances intratumoral delivery either by co-administration or covalent tethering, improving accumulation and efficacy of paclitaxel–PLGA nanoparticles, irinotecan silicasomes in PDAC, and gemcitabine in A549 xenografts; synergy with membrane-active peptides further augments ROS generation and mitochondrial depolarization. Conjugated iRGD micelles carrying platinum or camptothecin increased internalization, penetrated the BBB to reach glioma, and improved MRI lesion resolution. Clinically, the CEND-1 (iRGD) plus gemcitabine/nab-paclitaxel regimen is in phase 1 for PDAC (NCT03517176), with NRP-1 expression (median ≈ 60% across tumors) emerging as a stratification biomarker given survival differences (16.7 vs. 34.9 months for high vs. low NRP-1) [82].

Peptoid chemistry now extends ionizable-lipid design for mRNA therapeutics. A >200-member N-substituted glycine library (“Nutshells”) identified clusters with tunable organ tropism: cluster 8 favored liver, cluster 11 lung, governed by headgroup pKa and particle charge. Optimized formulations reproducibly formed <120 nm nanoparticles with >85% mRNA encapsulation; in vivo, peptoids 263/236 produced the highest anti-RSV antibody titers at 24 h, and Nutshell 236 delivered linear dose–responses (0.3–1.5 mg/kg^−1^), ≈2-fold higher titers than a DLin-MC3-DMA benchmark at 1.5 mg kg^−1^, sustained expression for five days, long-term stability at −80 °C (>6 months), and muted innate activation, demonstrating competitive or superior potency and safety for systemic mRNA delivery with peptoid ionizable components [83].

Within polypeptide carriers, elastin-like polypeptides (ELPs) built on (VPGXG)n motifs provide temperature-responsive self-assembly and ligand modularity to improve half-life, tumor retention, and intratumoral exposure across diverse cargos. Vascular-targeted NGR-ELP micelles increased CD13-mediated retention and extravasation; AP1 decoration enhanced IL-4R binding by ~10,000-fold; and F3-ELP-Dox nanoparticles raised tumor accumulation 4.2-fold and shrank tumors 3.8-fold versus non-F3 controls. Penetration and efficacy gains were observed with SynB1-ELP + hyperthermia (Taxol), R8-ELP in glioblastoma spheroids, and MMP-cleavable CPP-ELP–Dox overcoming resistance. Protein therapeutics (RGD-ELP-TRAIL, ELP-IFN-α, IFN-ELP(V) depots) achieved higher apoptosis, prolonged half-life, and reduced recurrence in resection models. Small-molecule delivery included paclitaxel–ELP nanoparticles with ~2× uptake over Abraxane and near-complete tumor control after a single dose, docetaxel in RGD-ELP with preferential cytotoxicity to breast cancer cells, and rapamycin targeting via FKBP12-ELP with ~3× mTOR suppression; nucleic-acid and radio-cargo delivery (TAT/AP1-ELP siRNA; 131I-ELP) broadened indications with improved biodistribution and safety [84].

**Figure 24:**
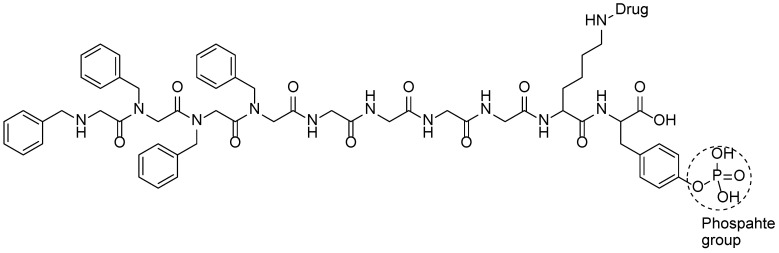
Peptoid-peptide hydrogelating template.

To secure long-acting small-molecule exposure, an in situ enzyme-responsive peptoid–peptide depot (Figure 24) was devised using a tetrabenzylamine–tetraglycine–D-lysine–O-phospho-D-tyrosine motif that covalently tethers drugs (e.g., zidovudine) via lysine esters. The lead sequence (NPhe)4GGGGk(AZT)y(p)-OH gelled subcutaneously within ~30 s (maturing ~90 min) at ≥2% *w*/*v*, formed narrow-radius fibers (≈0.78–1.8 nm), resisted proteolysis via D-residues/peptoids, and curtailed burst release relative to physical entrapment. In rats, a 5% *w*/*v* depot maintained zidovudine plasma levels within the IC_90_ window (30–130 μg/mL^−1^) for 35 days at clinically relevant exposure, establishing a protease-resistant, phosphatase-triggered platform for monthly dosing [85].

At the sequence-defined polymer frontier, polypeptoid amphiphiles enabled precision nanocarriers where side-chain chemistry dictates secondary structure and assembly. Amphiphilic block co-polypeptoids (30–80 nm micelles; CAC 0.002–0.03 mg/mL^−1^) remained stable for two months at 37 °C, encapsulated doxorubicin or camptothecin with up to 88% efficiency, and released payloads over 120 h with first-order kinetics. Aromatic N-phenylethyl substituents (30 mol%, block 50:20) maximized hydrophobic affinity and structural stability. Blank micelles were non-toxic (>90% viability); DOX-loaded micelles retained potency (IC_50_ ≈ 0.95 μg/mL^−1^ vs. 0.62 μg/mL^−1^ free DOX), enhanced intracellular fluorescence 2.7-fold, and in vivo reduced tumor volume by 71% versus saline and 54% versus free drug with minimal cardiotoxicity, linking backbone/side-chain design to self-assembly, loading capacity, and pharmacodynamics [86].

Self-assembling therapeutic hydrogels extend to hemopressin-derived polypeptides, where sequence substitution yielded FOK with the strongest β-sheet assembly, mechanical integrity, and pH sensitivity. FOK solutions (20 mg/mL^−1^) formed injectable, self-healing hydrogels (G′ > 1600 Pa; >95% encapsulation), releasing 68% Dox at pH 6.0 versus 23% at pH 7.4 over 168 h. In 4T1 cells, DOX/FOK achieved IC_50_ ≈ 1.54 μg/mL^−1^ (free 1.32 μg/mL^−1^) while blank gels were non-toxic (>90% viability up to 400 μg mL^−1^). Local injection suppressed tumor growth by 78.6% at day 7 versus 52.3% for free Dox, reduced cardiotoxicity, sustained tumor-site residence for >96 h, and maintained hydrogel integrity for six months under ambient storage, demonstrating durable, tumor-selective depot chemotherapy [87].

Ultrashort phosphorylated l-α and d-peptide hydrogels further offer rapid, enzyme-triggered implantation for long-acting antiretroviral delivery. Peptides bearing (naphthalene-2-yl)-acetyl-diphenylalanine-lysine-tyrosine motifs ester-linked to zidovudine formed soft gels (G′ ≈ 103–104 Pa; fiber radius ≈ 2 nm) within minutes upon phosphatase exposure; d-peptides resisted proteolysis for ≥28 days. Drug conjugation increased breakage strain up to 836%, enabling injectability and low burst. Subcutaneous dosing in rats sustained zidovudine within the IC_50_ range (30–130 ng mL^−1^) for 35 days, supporting monthly administration of small-molecule antivirals with tunable mechanics and release [88].

For active targeting, venom-derived polypeptides, notably the 36-mer chlorotoxin (CTX) from Leiurus quinquestriatus, function as precision ligands to glioma and other tumors via MMP-2, annexin A2, NRP-1, or chloride channel-binding. CTX-functionalized nanoparticles reproducibly boost tumor uptake and therapeutic indices: PEGylated iron-oxide–CTX probes enhance dual MRI/NIR contrast; CTX-guided iron-oxide carriers co-loaded with O6-benzylguanine potentiate temozolomide with a three-fold median survival extension in murine glioma; cyclodextrin–CTX systems deliver paclitaxel; hyaluronan-bridged CTX constructs carry gemcitabine; and CTX liposomes/niosomes deliver Dox or temozolomide. PAMAM-PEG-CTX vectors bearing TRAIL plasmids or siRNA accumulate selectively in glioma and prolong survival, positioning CTX and payloads such as temozolomide, Dox, paclitaxel, gemcitabine, anti-miR-21, and anti-survivin as a proven CNS-targeting toolkit. A phosphorylated D-peptide Napffky(p)G-OH platform was advanced for long-acting, multipurpose delivery of hydrophobic agents (MIV-150 and etonogestrel) via enzyme-triggered self-assembly into subcutaneous depots within ~10 s (maturation 198 min). The hydrogels exhibited shear-thinning injectability (viscosity 968.85 to 4.63 mPa·s from 1 to 1000 s^−1^), soft mechanics (G′ 583–4899 Pa), and outstanding biostability (>95% intact with proteinase K at 28 days; L-α controls degraded within 6 h). Covalent conjugation suppressed burst (72 h release: MIV-150 6.46%, etonogestrel 11.24%), with hydrolysis-driven kinetics (Weibull–Korsmeyer–Peppas, r2 > 0.9; n ≈ 0.9–1.0). In rats, plasma levels exceeded 4 × IC_90_ for MIV-150 (2.8 ng mL^−1^) and matched/exceeded Nexplanon-benchmark etonogestrel (≥0.153 ng/mL^−1^) for 49–56 days without histopathological toxicity, establishing a biostable, injectable D-peptide depot with tunable composition, mechanics, and release for long-term contraception and HIV prevention [89].

Polypeptides composed of natural amino acids achieve exceptional biocompatibility and stealth through sequences enriched with Pro, Ala, Ser, Thr, Gln, and Asn—uncharged, polar amino acids that promote hydration, while preventing folding. Their hydrophilicity arises from these neutral residues, which form dynamic hydration shells without introducing charges or aggregation-prone domains. Charged (Lys, Arg, Asp, Glu, His) and aromatic (Tyr, Trp, Phe) residues should be excluded, as they can mediate nonspecific interactions, trigger antigen presentation, or disrupt stealth. Their disordered structure avoids epitope formation and allows complete metabolic clearance without persistence in tissues.

## 6. Perspectives

Unlocking the full potential of these polymer families requires embracing broader chemical diversity than current paradigms typically allow. Instead of behaving as static coronas, future hydrophilic layers, whether derived from POx, zwitterionic motifs, or sequence-defined polypeptides, may adjust their hydration density, polarity, or segmental mobility as they encounter gradients in pH, ionic composition, or protein crowding. This could yield carriers that maintain stealth during circulation but reveal selective interaction domains upon tissue entry, introducing a new paradigm of ‘context-aware nanomaterials’. A comparison of the characteristics of the carriers in question can be made as follows Table 2.

Equally promising is the pursuit of chemical diversity as a driver of adaptive function. Many current platforms rely on well-established monomers or branching patterns, yet introducing noncanonical zwitterionic structures, unconventional POx monomers, or modular PG building blocks could dramatically expand the palette of physicochemical behaviors accessible to designers. By varying dipole geometries, aromatic content, or branching topology, researchers could explore how subtle shifts in electronic structure dictate hydration, protein interactions, and transport dynamics. Such studies would not replace existing materials but would elevate them, positioning each polymer family as a flexible framework rather than a fixed solution.

Future research should also explore how macromolecular behavior emerges across multiple length scales, from the organization of water molecules at the corona to the collective mechanics of nanoparticles navigating dense tissues. Different classes of materials already hint at this multiscale interplay: the high drug-loading cores achievable with POx, the multivalent surfaces of PGs, the immune-silent hydration of zwitterions, and the degradative precision of polypeptides. Integrating these strengths into unified design strategies, perhaps through hybrid architectures or responsive co-assemblies, may produce carriers that transition through several structural states as they move from bloodstream to interstitium to cell interior.

Another frontier lies in engineering temporal control, enabling materials to act not only in space but over time. Polymers capable of staged disassembly, sequential activation, or timed exposure of functional motifs could mimic biological logic, where processes occur in orchestrated sequences rather than continuous states. For example, a nanocarrier might travel through the bloodstream under a zwitterionic, protein-repelling shell; soften and shrink upon entering a tumor microenvironment; then reconfigure into a polycation–polypeptide hybrid that promotes endosomal escape. Such temporal choreography invites a new generation of materials that behave less like containers and more like programmable events.

Beyond the carriers themselves, future studies could embrace cooperative interactions between polymers and biological systems. Rather than navigating barriers in isolation, materials could transiently interface with endothelial cells, remodel local extracellular matrices, or respond to metabolic shifts, enabling a more deliberate progression through physiological checkpoints. This idea opens space for combining the osmotic adaptability of PG networks, the charge-switching potential of zwitterionic units, and the folding/degradation logic of polypeptides into coordinated delivery systems that operate in dialogue with tissues.

Achieving these ambitions requires a deeper integration of mechanistic insight into polymer design. The field has already demonstrated exceptional creativity in synthesizing diverse macromolecular architectures, but the next step demands rigorous frameworks that connect structural features to biological outcomes. Molecular dynamics simulations, machine-learned structure–activity relationships, and real-time imaging of corona evolution could help elucidate how monomer sequence, backbone rigidity, or dipole strength shape fate in vivo.

## 7. Conclusions

As the field of drug delivery drifts further from the long shadow of PEG, a new constellation of polymers begins to define the horizon, materials that no longer merely shield therapeutics but shape the very microenvironments through which they travel. In this shifting landscape, the search for a PEG successor has evolved beyond replacing a single molecule; it has become an exploration of how chemistry can sculpt biological fate with unprecedented subtlety. What follows is not just a comparison of polymer families, but a glimpse into the emerging logic of next-generation biomaterials: systems that blend molecular precision with biological intuition, and in doing so edge closer to the seamless integration of synthetic architecture within living systems.

Poly(2-oxazoline)s (POx) emerge as the most comprehensively validated PEG alternative. They combine excellent antifouling characteristics, low complement activation, broad biocompatibility at high systemic doses, and uniquely high drug-loading efficiencies, reaching up to ~45 wt% for paclitaxel and ~50 wt% for multidrug combinations. Their stimuli-responsive behaviors (pH-, temperature-, and LCST-switching), precise molecular weight control, and adjustable hydrophilic–hydrophobic balance enable programmable pharmacokinetics and controlled cargo release. Compared with PEG micelles, POx systems show superior stability against serum-induced disassembly and achieve deeper tumor penetration and prolonged circulation, particularly when stabilized by nucleobase crosslinking. These attributes position POx as a leading candidate for systemic chemotherapeutic delivery and nucleic-acid transport.

Polyglycerols (PGs) provide an orthogonal advantage through their chemically flexible dendritic architecture, enabling dense ligand presentation, pH-responsive linkers, and precise control of multivalency. Their inherent non-immunogenicity, hydrophilicity, and biodegradability support safe long-term use. PG-based systems excel in cell-type-specific targeting, as shown by mannose-decorated PG carriers achieving up to 88% uptake in infected macrophages, and in deep dermal penetration via β-CD–functionalized PG constructs. Their modular surface chemistry supports co-delivery strategies, pathogen-responsive release, and high drug solubilization, making PGs especially promising for infectious disease therapies, skin delivery, and intracellular antimicrobial strategies.

Zwitterionic polymers, including carboxybetaines, sulfobetaines, and phosphorylcholines, represent the closest biomimetic replacement for PEG’s hydration shell while avoiding its immunogenic liabilities. Their ultra-hydrated surfaces confer superior antifouling behavior, with no detectable anti-polymer antibodies, and their degradable backbones allow complete enzymatic breakdown, addressing PEG’s accumulation and toxicity risks. Demonstrated platforms, such as PLA-SB/PTX nanoparticles (19 nm), show full biodegradation, high drug stability, resistance to protein adsorption, and sustained-release profiles. These features make zwitterionic polymers uniquely suited for long-term implantation, repeated dosing regimens, and applications where immune neutrality is paramount.

Polypeptide-based carriers offer unmatched biological compatibility and degradability, with sequence-defined architectures and tunable secondary structures enabling precise control over circulation time and protease-mediated clearance. Their capacity to extend half-life by up to 60-fold without detectable immunogenicity, along with their inherent structural similarity to endogenous proteins, makes them ideal for protein–drug conjugates, enzymatic therapeutics, and applications demanding minimal long-term polymer burden. Sequence engineering further allows modulation of hydrophobicity, charge, and conformational stability to tailor release kinetics and biodistribution.

The future of targeted delivery may not hinge on discovering one dominant material, but on embracing a repertoire of polymers that behave less like passive carriers and more like intelligent participants in biological navigation.

## Figures and Tables

**Table 1:** Current FDA-approved PEGylated drugs.

Generic Name	Brand Name	FDA Approval Date
Pegfilgrastim	Neulasta^®^, Fulphila^®^, Udenyca^®^, Nyvepria^®^, Stimufend^®^, Fylnetra^®^	January 2002 June 2018 November 2018 June 2020 September 2022 May 2022
Peginterferon α-2a	Pegasys^®^	October 2002
Peginterferon α-2b	Pegintron^®^	December 2001
Peginterferon β-1a	Plegridy^®^	August 2014
Ropeginterferon α-2b	Besremi^®^	June 2021
Pegvisomant	Somavert^®^	March 2003
Pegvaliase	Palynziq^®^	May 2018
Certolizumab pegol	Cimzia^®^	April 2008
Pegaspargase	Oncaspar^®^	February 1994
Calaspargase pegol	Asparlas^®^	December 2018
Epoetin-β	Mircera^®^	November 2007
Rurioctocog-α-pegol	Adynovate^®^	November 2015
Damoctocog-α-pegol	Jivi^®^	August 2018
Turoctocog-α-pegol	Esperoct^®^	February 2019
Nonacog-β-pegol	Rebinyn^®^	May 2017
Pegloticase	Krystexxa^®^	September 2010
Pegcetacoplan	Empaveli^®^ Syfovre^®^	May 2021 February 2023
Zilucoplan	Zilbrysq^®^	October 2023
Avacincaptad pegol	Izervay^®^	August 2023
Pegunigalsidase-α	Elfabrio^®^	May 2023
Eflapegrastim	Rolvedon^®^	September 2022
Lonapegsomatropin	Skytrofa^®^	August 2021
PEGylated liposomal doxorubicin	Doxil^®^/Caelyx^®^	November 1995
PEGylated liposomal irinotecan	Onivyde^®^	October 2015
Patisiran	Onpattro^®^	August 2018
BNT162b2 mRNA vaccine	Comirnaty^®^	August 2021
mRNA-1273 vaccine	Spikevax^®^	January 2022
Naloxegol	Movantik^®^	September 2014
Polidocanol	Asclera^®^	March 2010

**Table 2:** Comparison of PEG with proposed polymer alternatives.

	PEG	POx	PG	Zwitterionic	Polypeptide
Circulation time	++	+	+	++	++
Loading capacity	-	++	+	+	+
Degradability	-	0	+	++	++
Immunogenicity	-	+	++	++	++

The qualitative scoring is inherently subjective and is derived solely from the data reported in the references discussed in this manuscript.

## Data Availability

All data are available in a public, open-access repository.
